# Accelerating *Campylobacter* zoonosis in the Anthropocene

**DOI:** 10.1073/pnas.2609969123

**Published:** 2026-07-27

**Authors:** Oakem J. Kyne, Bridget S. Penman, David J. Kelly, Samuel K. Sheppard

**Affiliations:** ^a^ Ineos Oxford Institute for Antimicrobial Research, University of Oxford, Oxford OX1 3EL, United Kingdom; ^b^ Department of Biology, Life and Mind Building, University of Oxford, Oxford OX1 3EL, United Kingdom; ^c^ School of Biosciences, The University of Sheffield, Sheffield S10 2TN, United Kingdom

**Keywords:** pathogen, intensive agriculture, evolution, *Campylobacter*, poultry

## Abstract

Intensive poultry farming has transformed global bird populations, concentrating billions of chickens into dense industrial systems that fundamentally reshape ecological space. As birds are important pathogen reservoirs, these changes can have important consequences for the spread of zoonoses including *Campylobacter jejuni*—the leading cause of bacterial gastroenteritis. Analyzing 2,747 genomes from chickens and wild birds, we show that poultry intensification has eroded historic host–strain associations and created a new and expanding ecological niche. Phylogenetic reconstructions reveal an estimated 100-fold increase in chicken-to-wild-bird host transitions since 1900 compared to predomestication levels, alongside dramatic post-1960 expansions of chicken-associated lineages and rising pathogen effective population sizes. Model simulations further indicate that expanding, high-density chicken populations could act as ecological “pathogen sponges,” absorbing and amplifying diverse strains while sustaining high prevalence and coinfection rates. Genome-wide association analyses show that strains adapted to the chicken niche frequently acquire genes linked to oxidative stress, metal homeostasis, motility, and antimicrobial resistance. Together, these findings demonstrate that intensive poultry farming is reshaping pathogen ecology and accelerating the evolution and spread of a major zoonotic bacterium, with direct implications for human health.

## Significance

Human-driven environmental change in the Anthropocene is reshaping how pathogens emerge, evolve, and spread. This study shows that the global expansion of intensive poultry farming has created a ecological system that fundamentally alters interactions between hosts and microbes. Using large-scale genomic data, we reveal that dense, rapidly growing livestock populations can promote cross-species transmission, sustain high pathogen diversity, and accelerate adaptive evolution, including traits linked to persistence and antimicrobial resistance. These findings may be relevant beyond a single pathogen system and demonstrate how modern food production can generate new evolutionary dynamics with broad consequences. Understanding these processes is essential for predicting and mitigating the emergence of infectious diseases in an increasingly human-dominated world.

Human activity is driving epoch-defining change in the earth’s biosphere (1). In recent decades, trends in human food consumption have led one bird species to dominate over all others. Chickens now comprise 70% of all bird biomass on earth (2). The domestication of this species from red jungle fowl [*Gallus gallus* (3)] may have begun in Southeast Asia as far back as 8,000 y ago (4). However, the rise of poultry is largely a modern phenomenon with the number of chickens increasing sevenfold since the 1960s to roughly 31 billion birds today (5).

For domesticated jungle fowl, life on the farm is a dramatic departure from the ecology of ancestral populations. Huge, genetically homogeneous, flocks are held at high stocking densities and fed a uniform diet throughout their relatively short lives. While this imposes challenges to animal health and welfare (6, 7), the global rise of chickens could be viewed as evolutionary success by some measures. However, the impact of this ecological transformation does not end here. The chickens themselves are a niche for the numerous microbes that live within and upon them.

Although most of the chicken microbiome comprises harmless commensal organisms, chickens are also implicated in the spread of zoonotic pathogens (8, 9). Among these is one of the world’s most common causes of bacterial gastroenteritis, *Campylobacter*. The predominant pathogenic species, *Campylobacter jejuni* and *Campylobacter coli,* are found in extremely high numbers in the ceca of healthy chickens (10–12) and infect humans principally via fecal contamination of food, especially poultry (12). In fact, poultry are the foremost reservoir for human infection with an estimated 60 to 80% of cases attributable to isolates originating in poultry (13–19). As global poultry numbers increase, so do the opportunities for environmental spillover into the human food chain (20–22). However, while the epidemiology of zoonotic transmission to humans is well understood (23), little is known about how the expansion and spread of the chicken niche affects the evolution and transmission of enteric bacteria between wild and domesticated host species.

Wild birds are a natural reservoir for *Campylobacter*, in particular *C. jejuni*. Genomic analysis of strain variation has revealed differentiation among lineages inhabiting a particular bird host species (24–30). A simple explanation for this is that *C. jejuni* are microaerophilic and poorly adapted to life outside the host (31). Over time, independent evolution in genetically isolated populations has given rise to ecological “specialists” (22, 32). However, this simple model of niche stratification is not reflected in all *C. jejuni* populations. This is most obvious in chickens where a single flock, or even a single bird, can simultaneously harbor multiple *C. jejuni* lineages (30, 33)

Cohabitation of multiple sympatric strains presents an evolutionary quandary as the fittest adapted strains might be expected to outcompete others. This is especially true where high stocking densities facilitate transmission and very large population sizes increase the efficiency by which natural selection favors livestock-adapted genotypes (34). This explanation, however, does not take full account of the enormous scale of ecological change in intensive livestock production. In such rapidly expanding niche space, selection underlying adaptation and competitive exclusion is balanced against continued colonization by new strains. As the niche continues to grow and spread there are ever more opportunities for host transitions from wild animal and bird species (31, 35), indeed several *C. jejuni* strains have been isolated from multiple species (32, 36). These ecological generalists (27, 32, 37) indicate recent host transitions (37) and nowhere is this more evident than in chickens.

While other reservoirs including cattle, pigs, companion animals, and environmental water sources contribute to *C. jejuni* transmission ecology, we focused specifically on wild birds and chickens because *C. jejuni* is considered a strongly avian-associated organism (27, 31). Wild birds likely represent an important ancestral reservoir, whereas chickens constitute a recently expanded anthropogenic niche characterized by extremely high host density and transmission intensity (22, 31). Focusing on these hosts allowed us to investigate avian *C. jejuni* host adaptation and spillover dynamics within a biologically coherent framework while minimizing additional ecological and physiological heterogeneity associated with other hosts (22, 27, 31).

Understanding how increasingly intensive farming practices and global livestock trade networks influence the emergence of zoonotic pathogens is critical to safeguarding human health. Each year, around 550 million people—including 220 million children under five—suffer from foodborne diarrhea, with *Campylobacter* being the most common bacterial cause (38). To address this growing public health threat, we analyzed the genomes of 2,747 *C. jejuni* isolates from chickens and wild birds to identify host-associated genes and genotypes and to quantify how major shifts in livestock host niches drive pathogen evolution. We further describe how changes in effective population size influence evolutionary trajectories and host transition rates and identify specific genetic adaptations that may enable strains to proliferate in chickens, offering insight into how agricultural intensification can accelerate the emergence of zoonotic pathogens.

## Results

### *C. jejuni* Lineages Display Host Generalist and Host Specialist Ecology.

**Fig. 1.**
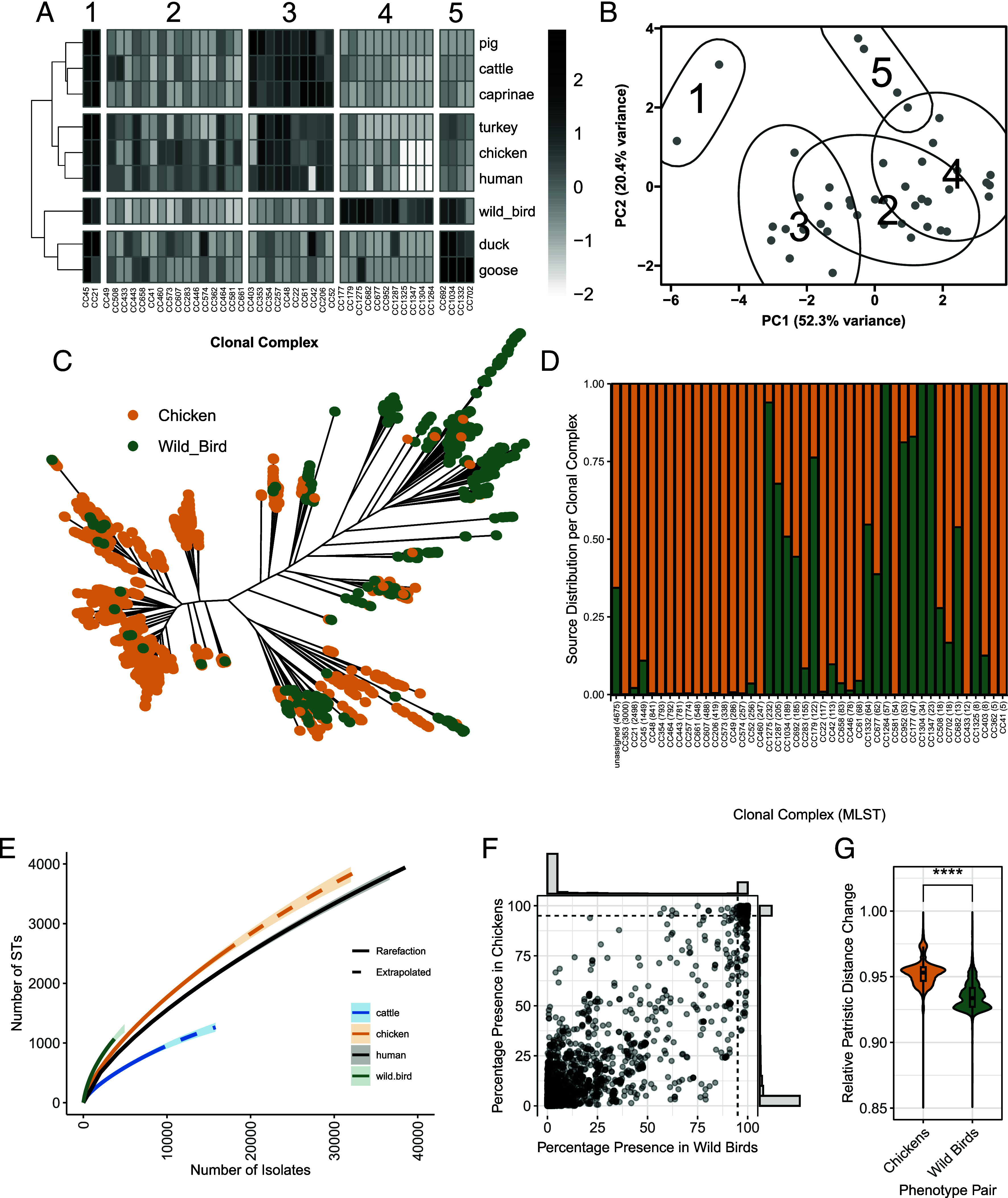
Genetically diverse *C. jejuni* populations have varying levels of host specificity. (*A*) Heatmap showing host association of 43 major MLST clonal complexes (CC), separated into five clusters based on normalized host distributions (log-transformed z-score). (*B*) Principal component analysis of host distributions with each point representing a CC. CC complex clusters are consistent across both the heatmap (*A*) and the PCA (*B*). (*C*) Unrooted RAxML core genome phylogenetic tree showing the distributions of wild bird and chicken isolates. (*D*) Host source segregation of isolates belonging to different CCs. Some CCs are more specific to chickens or wild birds, while others are found in both groups, indicating host generalism. (*E*) Rarefaction analysis of ST discovery for samples collected from cattle, chicken, human, and wild bird hosts, demonstrating considerable diversity in wild birds and chickens. (*F*) Pangenome gene presence–absence comparison between wild bird and chicken isolates, showing incomplete gene pool segregation across both hosts. (*G*) Recombination analysis using ClonalFrameML showing how much of the phylogenetic distances in the RAxML tree is a result of recombination. This shows that more recombination was detected in chicken isolates than in wild bird isolates.

Consistent with many pathogenic bacteria, genotype variation in *C. jejuni* can be considered by comparing allelic variation at seven house-keeping loci that define the multilocus sequence type (ST) (39). Comparing isolate source data and *C. jejuni* clonal complexes (CC) (STs sharing four identical alleles) revealed host-associated clustering, with some genotypes associated with a single host (specialists) and others exhibiting generalist ecology as previously described (32) (Fig. 1*A*). Isolates from waterfowl segregated from other wild birds, forming two discrete clusters. Weaker host partitioning was observed among other hosts that broadly divided into two groups: farmed mammals (pigs, cattle, sheep, and goats) and farm poultry (chickens, turkeys) and humans. The grouping of human isolates with farmed poultry is likely because human infection is often (60 to 80%) caused by isolates from this reservoir (13–19). Quantitative clonal complex clustering revealed five groups, with Ward’s hierarchical algorithm. Group 1 contained the host generalist lineages ST-45 and ST-21, while groups 4 and 5 were strongly associated with wild birds and waterfowl, respectively. Groups 2 and 3 showed weaker host associations, spanning farmed mammals, poultry, and humans. Except for groups 4 and 5, CC groupings were not phylogenetically distinct (*SI Appendix*, Fig. S1) and showed only partial separation in multivariate space (PCA; Fig. 1*B*). This provides initial evidence for *C. jejuni* transmission among different host populations.

### Chickens Have Been Colonized by Multiple Wild Bird Lineages.

The phylogeny of 2,747 *C. jejuni* isolates from chickens (n = 1,892) and wild birds (n = 855) shows strains from both sources are widely distributed across the tree (Fig. 1*C*). Specialization of *C. jejuni* to particular host populations is typically reflected in closely related genotypes that form distinct clusters on the phylogeny, each corresponding to the host population from which they were sampled. In isolated wild bird populations, *C. jejuni* tends to evolve more independently, and over time genetic drift produces host-associated lineages (40). Deviations from this null expectation indicate recent host transitions. Comparing host distribution patterns of both tree sequence clusters and isolate clonal complex segregation (Fig. 1*D*) revealed *C. jejuni* lineages exclusively associated with single host populations (specialists), as well as lineages found in multiple hosts, consistent with recent colonization. Overall, this suggests that hosts have been repeatedly exposed to diverse *C. jejuni* lineages, potentially linked to the emergence of host generalism in the pathogen (37).

To understand the trends in *C. jejuni* diversity in different hosts we analyzed the numbers of STs (Fig. 1*E*), explored the patristic distance (tree branch length) (Fig. 1*G*), and quantified the pangenome similarity for isolates from different hosts (Fig. 1*F*). The greatest number of *C. jejuni* STs were observed among isolates from chicken and human sources but rarefaction analysis of STs showed that wild bird *C. jejuni* (Shannon = 614 ± 92.2; Simpsons = 208 ± 43.4) have the most clonal diversity for a given number of samples, compared to chickens (Shannon = 321 ± 23.6; Simpsons = 71 ± 4.5), humans (Shannon = 222 ± 11.6; Simpsons = 48 ± 2.0), and cattle (Shannon = 69 ± 6.3; Simpsons = 24 ± 1.3). Consistent with this, the average patristic distance between all wild bird isolates was significantly larger than between chicken isolates, 0.13 and 0.059 substitutions per site respectively (Wilcoxon rank-sum test, W = 8.86 × 10^10^, *P* < 2.2 × 10^−16^). Finally, the *C. jejuni* core genome size (90%) was similar among hosts (Fig. 1*F*), 1,371 in chickens and 1,330 in wild birds (1,315 shared core genes). While wild birds had a larger accessory genome (<5%), 4,917 genes compared to 3,787 genes in chickens, with 60% (2,943) of accessory genes shared between host populations.

It is expected that wild birds harbor greater strain diversity as they comprise multiple host species with associated *C. jejuni* evolving in allopatry (25, 40). However, chicken hosts also harbor considerable lineage diversity indicative of rapid within host evolution, colonization by strains from other sources, or both. Genotype diversity, phylogenetic clustering, and pan-genome overlap all suggest degradation of a clear phylogenetic signal of host population segregation. This is consistent with a scenario where the global spread and increase in chicken population size increased opportunities for historically isolated wild bird *C. jejuni* strains to colonize chickens. This enhanced niche overlap among *C. jejuni* strains increases opportunities for horizontal gene transfer among lineages in the same host individual. Some evidence in support of this was provided by the significantly higher (Wilcoxon rank sum test, *P* < 2.2 × 10^−16^) recombination amounts detected among isolates from chicken (Fig. 1*G*). While elevated recombination rates in chicken-associated isolates may reflect biological differences linked to intensive livestock production and increased transmission opportunities, we cannot exclude the possibility that differences in sampling density and population structure between host groups influence these patterns.

### Genome-Wide Association Studies (GWAS) Reveal Chicken Niche Adaptations.

**Fig. 2.**
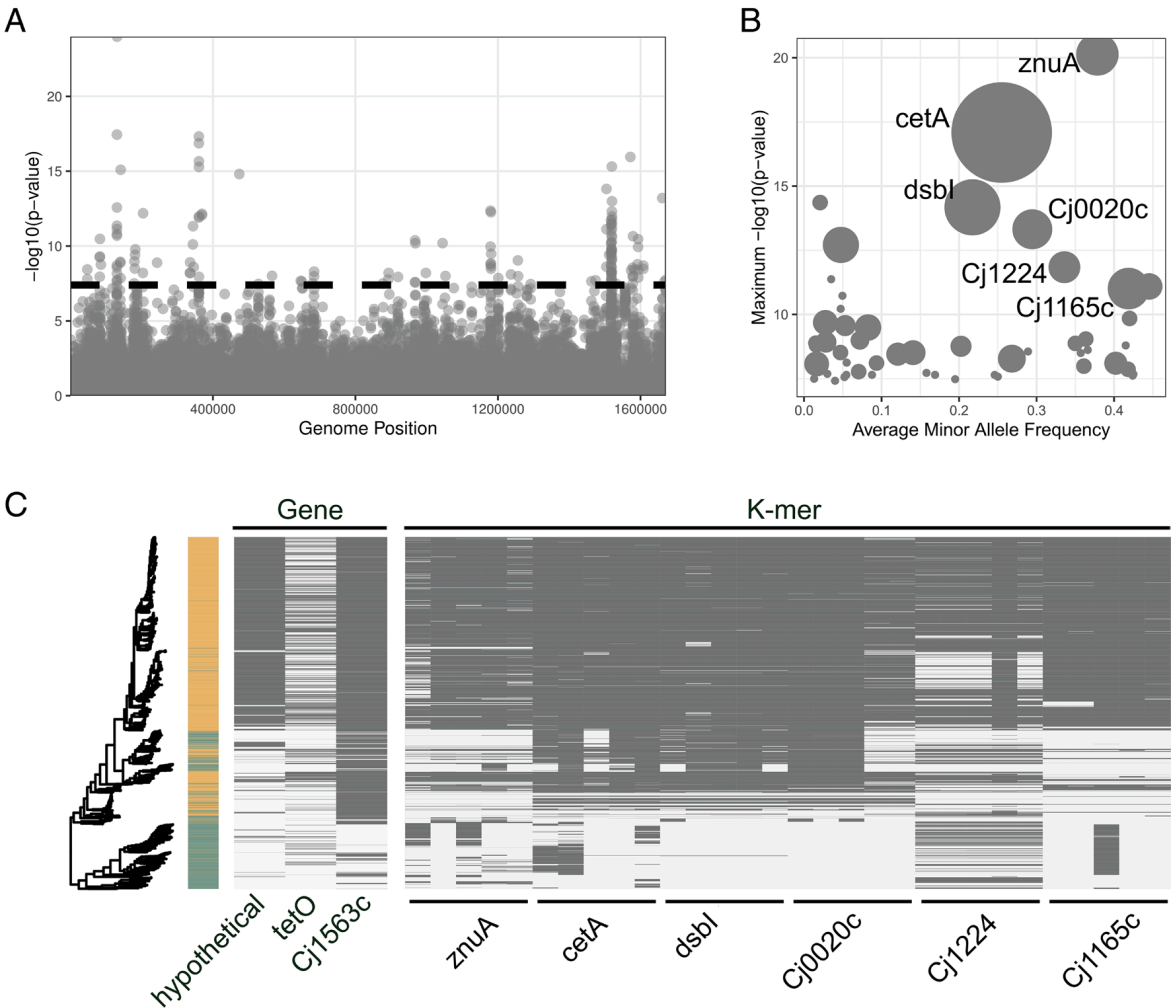
Genome-wide association analysis identifies *C. jejuni* genes and k-mers associated with chicken colonization. (*A*) Manhattan plot of k-mer associations from the GWAS mapped to the reference genome of an ST-21 chicken isolate (BioSample: SAMEA3556520). The dashed line indicates the genome-wide significance threshold (*P*-value < 4.01 × 10^−8^); k-mers exceeding this threshold are shown above the line. (*B*) Aggregated gene-level hits derived from significant k-mers, plotted as minor allele frequency (MAF) vs. −log_10_ (*P*-value). Labeled genes meet the following criteria: MAF > 0.05, >10 significant k-mer hits, and *P*-value < 4.01 × 10^−8^. (*C*) Distribution of significant k-mer hits (*P*-value < 4.01 × 10^−8^) and significant gene-level hits from the gene-based GWAS (*P*-value < 7.1 × 10^−6^) mapped onto a maximum-likelihood phylogeny inferred with RAxML from 2,747 isolates from chickens (yellow) and wild birds (green). The phylogenetic distribution of hits across chicken-associated lineages suggests convergent evolution and/or horizontal gene transfer within the chicken host niche.

Following spillover into a new host population, a pathogen may adapt to the novel host species (35). This process can leave genomic signatures associated with the new host population, potentially revealing the evolution events linked to the colonization of farmed chickens from wild bird hosts. To identify genomic features associated with chicken derived *Campylobacter* isolates, we performed GWAS (Fig. 2). This identified chicken associated core and accessory genome variation in homologous and nonhomologous sequence. Initially we calculated narrow sense heritability (h^2^) for our samples, this gave h^2^ as 0.89 indicating strong genome–phenotype associations, which is desirable for GWAS studies.

GWAS identified three genes that were significantly associated with chicken isolates after stringent filtering (adjusted *P*-value < 7.1 × 10^−6^, 0.05 < allele frequency < 0.95, positive β values) (Fig. 2*C*). These included i) *tetO*, encoding a ribosomal protection protein that confers tetracycline resistance (*P*-value = 3.91 × 10^−7^); ii) *cj1563c*, encoding a MerR-like transcriptional regulator (*P*-value = 2.78 × 10^−6^); and iii) a gene encoding a hypothetical protein containing a potential ubiquitin binding (UBA) domain (*P*-value = 3.43 × 10^−7^). In addition to genes that were more likely to be present in isolates from chickens, there was also evidence of host-associated homologous sequence variation (Fig. 2). Specifically, k-mer–based GWAS using a linear mixed model revealed six loci with chicken associated alleles (*P* < 4.01 × 10^−8^; 0.05 < MAF < 0.95; k-mer count ≥ 10). These were i) *znuA* (k-mer hits = 22; *P*-value = 7.38 × 10^−21^); ii) *cetA* (k-mer hits = 153; *P*-value = 8.15 × 10^−18^); iii) *dsbI* (k-mer hits = 42; *P*-value = 6.71 × 10^−15^); iv) *cj0020c* (*docA*/*ccpA1*) (k-mer hits = 19; *P*-value = 4.8 × 10^−14^); v) *cj1224* (*herB*) encoding a hemerythrin (k-mer hits = 11; *P*-value = 1.44 × 10^−12^); and vi) *cj1165c* encoding a putative amino acid exporter (k-mer hits = 21; *P*-value = 9.81 × 10^−12^); k-mer results in *SI Appendix*, Table S1.

Several genes enriched in chicken-associated *Campylobacter* isolates can be considered in the context of selective pressures within poultry production environments. The *tetO* gene confers tetracycline resistance by encoding a ribosomal protection protein that displaces tetracycline from the ribosome (41). Its enrichment in chicken isolates is potentially explained by the widespread use of antimicrobials in poultry production, in contrast to the low antibiotic exposure typical of wild bird populations. The prevalence of *tetO* in our isolates also corresponds with sampling year as isolates since 2015 have much higher *tetO* prevalence (*SI Appendix*, Fig. S6). Additional AMR-related loci included *cj1563*, encoding a putative copper-homeostasis regulator (42) (although its specific function remains uncharacterized) and a UBA-domain protein of unknown role in host adaptation.

Genes involved in metal acquisition also showed strong host-specific signatures. The most significant k-mer association corresponded to *znuA* (*P*-value = 7.38 × 10^−21^), which encodes the high affinity periplasmic binding component of the ZnuABC zinc transporter essential for zinc uptake and crucial for chick colonization (43–45). Its enrichment in chicken isolates could indicate adaptation to zinc-limited conditions or heightened zinc requirements in the poultry gut. Conversely, significant k-mer associations were detected in *zupT* (*P*-value = 1.97 × 10^−16^), a low affinity zinc transporter (43), in wild bird isolates. This reciprocal pattern suggests that chicken-associated lineages may experience stronger zinc limitation, favoring high-affinity uptake systems, whereas isolates from wild birds may encounter more accessible zinc.

Several chicken-associated *Campylobacter* loci were linked to oxidative stress tolerance. These included *herB* (*cj1224*), encoding a hemerythrin; *dsbI*, encoding a disulfide bond formation enzyme; and *docA/ccpA1*, encoding a cytochrome C551 peroxidase. Together, segregating variation in these genes may indicate changes to oxidative stress and exposure in the poultry gastrointestinal environment.

Motility-related selection was also evident. The GWAS identified *cetA*, encoding an Aer-like chemotaxis energy transducer. In partnership with CetB, CetA links changes in redox state to flagellar motility (46), likely via the electron transport chain. As chemotaxis is critical for locating favorable niches in the avian gut, the enrichment of certain *cetA* alleles in chicken isolates suggests that motility regulation may be involved in host-specific adaptation.

Finally, chicken associated sequence variation within the *cj1165* gene, indicates potential selection on the ThrE family of solute exporters and amino acid efflux systems. ThrE exporters regulate intracellular levels of dicarboxylates and amino acids such as L-threonine and L-serine (47, 48). L-serine and various peptides serve as preferred carbon and nitrogen sources for *C. jejuni* (49, 50). Increased reliance on these nutrients in the chicken intestinal niche may therefore favor strains with more effective efflux control to prevent toxic amino acid accumulation. Taken together, the genes highlighted across the different GWAS analyses suggest contrasting host environments could impose distinct selective pressures, driving both gene presence and allelic adaptations in *Campylobacter* lineages. While these genomic loci are strong candidates for adaptation to the chicken niche, further experimental studies would be required to determine their individual contributions.

### The Global Rise of Poultry Coincides with the Emergence and Spread of Chicken Adapted *C. jejuni*.

**Fig. 3.**
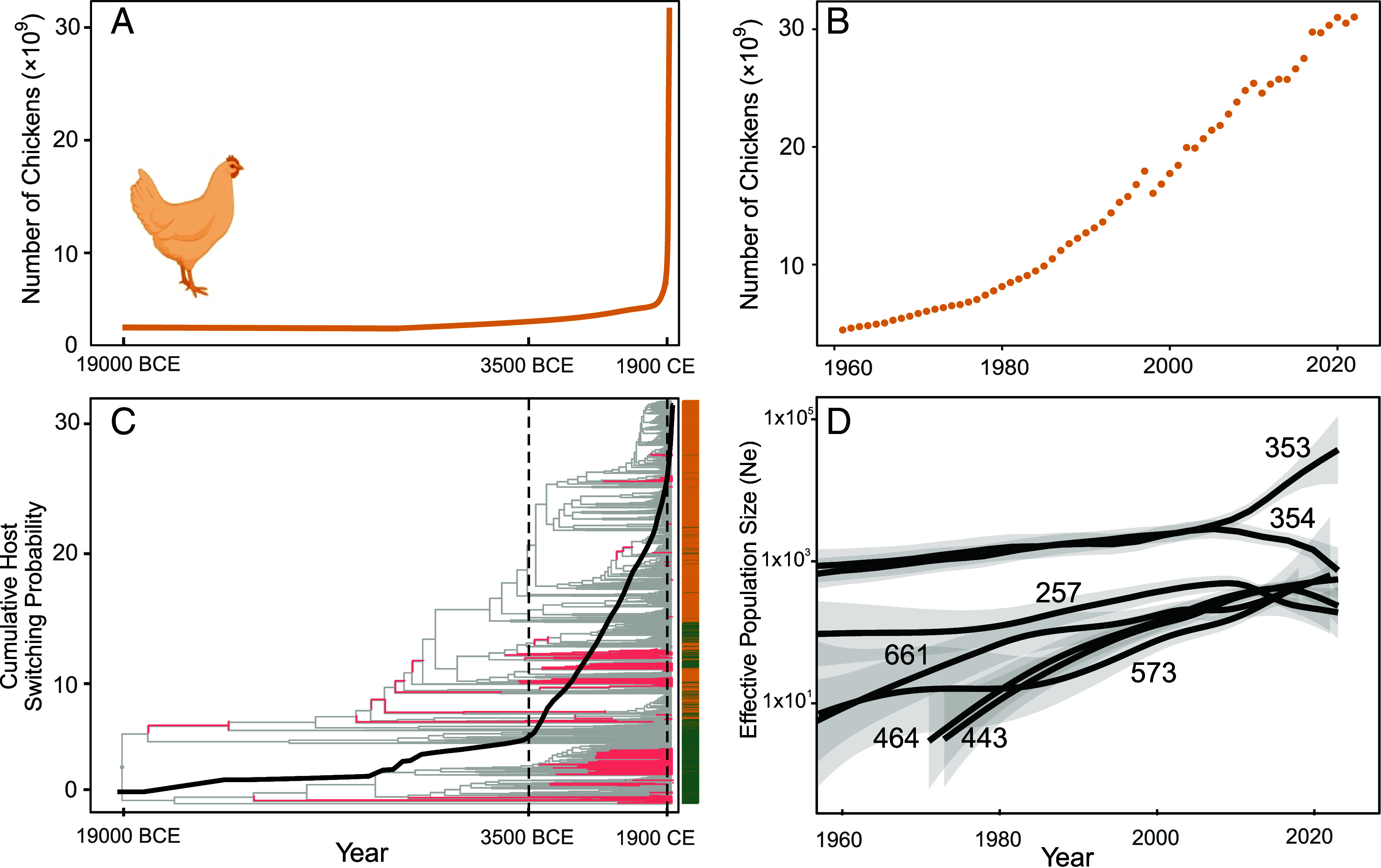
Chicken population expansion is associated with increased pathogen population size and elevated host transition rates. (*A* and *B*) Temporal increase in the global chicken population size, illustrating rapid expansion associated with the intensification of modern poultry farming. Data were obtained from FAOSTAT and historical population estimates. (*C*) Cumulative host transition events inferred using TreeBreaker and mapped onto a dated maximum-likelihood phylogeny reconstructed with RAxML. Dashed vertical lines indicate approximate timings of major milestones in chicken history, including domestication (~3500 BCE) and the onset of industrial-scale intensification (~1900 CE). The accumulation of inferred host transition events (black line) increases over time and parallels the expansion of the global chicken population. (*D*) Effective population size (N_e_) trajectories for seven chicken-associated CC, with six showing significant increases in N_e_ over time. These demographic trends coincide with the documented growth in global chicken population size.

Since the 1960 s the chicken population has increased sevenfold to a staggering 31 billion (5) (Fig. 3*A*). This represents a huge change in niche size for *C. jejuni* and changes the contact rate and transmission rate between chickens and wild birds. This increase in chicken host niche space corresponded with an increase in the effective population size of chicken specialist *C. jejuni* strains over the same period. All analyzed CC showed significant increases in effective population sizes with large CI (Fig. 3*D*) except for clonal complex 354, which showed a fold change of 0.79 (95% CrI: 0.23 to 2.7). Clonal complex 257 showed the most modest increases, with fold changes of 2.0 (95% CrI: 0.32 to 12). The five other common chicken associated CC displayed significantly larger increases in effective population sizes with a 180-fold change (95% CrI: 20 to 1,600) for CC-443, an 81-fold change (95% CrI: 9.2 to 700) for CC-464, a 72-fold change (95% CrI: 3.7 to 1,400) for CC-661, a 48-fold change (95% CrI: 10 to 230) for CC-353 and a 48-fold change (95% CrI: 3.9 to 590) for CC-573 (Fig. 3*D*). There were insufficient numbers of dated samples in wild bird CC to investigate potential changes in effective population size.

Chicken domestication and population increase were also associated with increased transition rates between host species (Fig. 3*C*). Host species transition events were inferred across the phylogeny using the Bayesian inference tool treeBreaker. There is some debate about the timescale of chicken domestication. Inference from genome data and lineage divergence is consistent with domestication between 6,000 and 8,000 BCE, but lineage divergence may predate the start of domestication (51). By contrast, the oldest directly datable archaeological domestic chicken remains are more recent (1650–1250 BCE) (52).

Low initial rates of *C. jejuni* host transition were observed between the tree root and 3500 BCE (an intermediate domestication estimate; gradient = 0.00029 transition/year). A step change occurred between 3500 BCE and 1900 CE (gradient = 0.0043 transitions/year) with the rate 15.0 times higher than predomestication levels (gradient = 0.031 transitions/year). The most striking increase in host transition rates occurred from 1900 CE to the modern day. This coincides with modern intensification of poultry production, and the inferred rate of *C. jejuni* host transition was more than 100 times higher in this period compared to predomestication levels. While there are potential biases due to uneven historical sampling, estimates were relatively stable when accounting for root date CI, with estimated increases of 12.1 to 16.5-fold for the preintensification period, and 86.9 to 119-fold for the intensification period, both relative to predomestication levels.

### Chicken Population Size Drives *Campylobacter* Host Transition Dynamics.

**Fig. 4.**
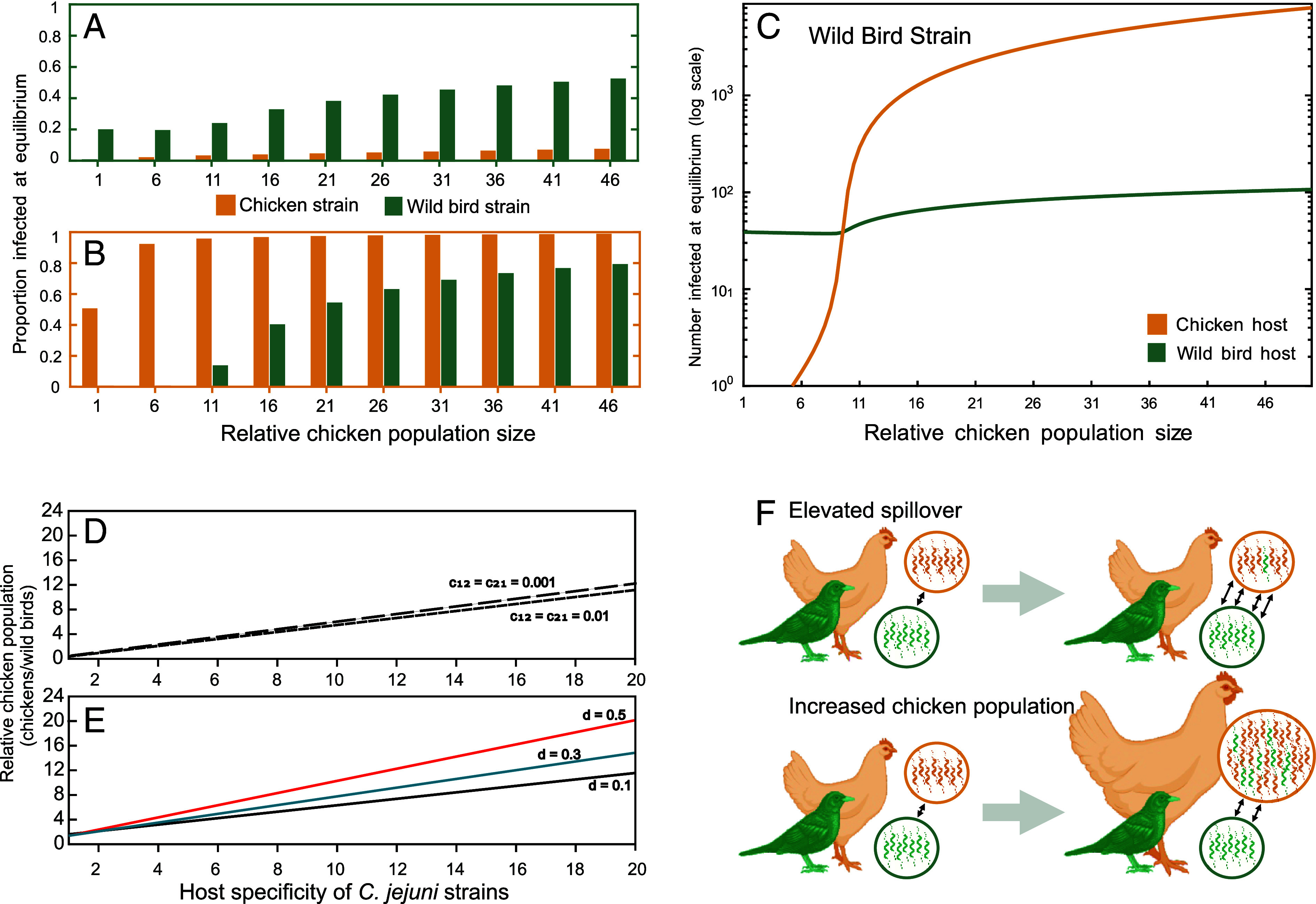
Host population size drives zoonotic transition of wild bird strains to chickens. Mathematical modeling demonstrates the importance of chicken population size for host transition dynamics for wild-bird and chicken specialist *Campylobacter* strains. (*A*) The proportion of wild bird hosts with chicken and wild bird strains for different chicken population sizes. (*B*) The proportion of chicken hosts with chicken and wild bird strains for different chicken population sizes. In both hosts, infection levels rise with chicken population size. At high chicken population sizes, chickens also carry a substantial burden of wild-bird-specialist strains. (*C*) Absolute number of chickens and wild birds infected with wild-bird-specialist strains across varying chicken population sizes. After the lines intersect, the transition point, there are more chickens infected with wild bird strains than there are wild birds. (*D* and *E*) Sensitivity analysis of parameters influencing the transition threshold, defined as the chicken population size at which the number of chickens infected with wild bird specialist strains exceeds the number wild birds infected with these strains. The threshold depends on host specificity (k), which represents how much more efficiently the strains transmit within their preferred host species. (*D*) Increasing cross species contact rates (c12, c21) has relatively little effect on the transition threshold across different levels of host specificity (k). (*E*) Increasing the coinfection penalty (d), defined as the reduction in pathogen shedding during coinfection relative to single infection, substantially increases the transition threshold across different levels of host specificity. (*F*) Conceptual summary of modeling results. Increasing the contact rate (elevated spillover) has minimal effect on the host transition. Increasing chicken population size elevates infection burden in both host species and promotes spillover and establishment of wild-bird-specialist strains in chickens.

A change in the number of hosts can influence how bacteria spread within and between host species. As a host species becomes more abundant, greater numbers of bacteria are shed into the environment, increasing opportunities to infect new hosts. However, bacteria typically colonize their original host species, to which they are adapted, more efficiently than new hosts (24, 53–55). Consequently, shifts in host population size influence host transitions through a balance of contact rates (which create opportunities for transmission) and bacterial host specificity (which constrains successful colonization). To investigate how the global rise of chickens may have affected this balance, we developed an epidemiological model simulating *C. jejuni* dynamics in linked chicken and wild bird populations (Fig. 4).

We initially parameterized the model to reflect prepoultry-intensification conditions, where wild bird and chicken populations were assumed to be of similar sizes. Although chickens had a shorter life span and greater rates of within-species contact than wild birds, *C. jejuni* strains principally circulated within the host populations for which they were specialized, despite some contact between the host populations (Fig. 4 *A* and *B*). As the chicken population size increased, it reached a critical point at which the host barrier broke down (Fig. 4 *A*–*C*). Once chicken populations exceeded this threshold, wild bird strains became self-sustaining in chickens, with the number of chicken infections with the wild bird strain rapidly surpassing those in wild bird hosts. This represents an evolutionary tipping point for the wild bird associated *Campylobacter* strains and occurred in our model despite a 10-fold transmission disadvantage for strains in the host species to which they were not adapted. This increasingly positioned chickens as a major reservoir for wild bird strains.

This quantitative change in the major host reservoirs describes a chicken population size at which the number of chicken infections with wild bird strains equals the number of wild bird infections with wild bird strains. This is a potential evolutionary tipping point, above which one might expect the predominant selective pressure acting on wild bird strains to be the ability to survive within chickens, not wild birds. In our model, the size of the chicken population required for this putative tipping point was linearly related to the ratio of host preference between the strains, i.e. how many times more likely the wild bird strain was to infect wild birds than chickens (parameter k, Fig. 4 *D* and *E*). The gradient of this relationship depended on the assumed level of competition between the strains in a coinfected bird, i.e. the reduction in transmission of both individual strains from a coinfected bird (parameter d), and to a lesser extent on the connectedness of the host populations (*c_12_* and *c_21_*).

Increasing connectivity between chicken and wild bird populations (elevated spillover) had limited effect on the tipping point (Fig. 4*D*), but increasing the penalty for coinfection greatly increased the relative population size of chickens required for the transition point (Fig. 4*E*). This suggests that host population size, rather than between-host population exposure, could be the primary driver of niche invasion (Fig. 4*F*). Furthermore, despite the strains each having a preferred host species in which they transfer more efficiently, in a sufficiently large chicken population even poorly adapted wild bird *C. jejuni* were able to proliferate. This potentially favors ecological generalist rather than specialist lifestyles (32).

## Discussion

Anthropogenic ecosystem change can lead to spill-over of zoonotic pathogens into new host populations (56, 57). The rise of intensive livestock production has led to the emergence of a vast host niche in which members of the microbiome can proliferate and spread through global food production networks. For pathogens such as *C. jejuni* this represents an infection risk to humans. For example, chickens shed around 1.8 × 10^18^ to 3.2 × 10^20^ CFU of *Campylobacter* per day [31 billion chickens × 0.5 to 0.9 infection levels (58) × 115 g fecal matter/day (59) × 10^6^ to 10^8^ CFU/g of feces (60)], and these can infect humans, principally through contaminated food (12). Because of the sheer magnitude of *Campylobacter* entering the food production chain, perhaps it is no surprise that 60 to 80% of human infections originate in the poultry reservoir (13, 17, 19, 61). While this is something of an oversimplification, there is an urgent need to address the role of industrialized livestock production in human infection.

Global expansion of poultry production has created opportunities for zoonotic transmission among previously isolated bird populations. *C. jejuni* is common in many wild birds with prevalence varying by species, diet, and season (31, 40, 62–64). Wild birds are thought to be an important source of chicken infection by either gaining access to housed chickens or by contaminating accessible surface water or soil (65). Consistent with previous studies, we found that different wild bird species harbored genetically distinct host specialist *C. jejuni* populations as well as lineages found in multiple hosts (24, 25, 27–30, 66, 67). These host association patterns could be explained by strain-specific differences in host colonization potential. For example, in in vivo infection experiments, isolates from a given host species will more readily colonize other individuals of the same species (24). However, strains can evolve quickly, and studies have shown that several fecal-oral passages through chicks greatly improve colonization potential of *Campylobacter* strains (68). Therefore, multiple exposures and recent host transitions could explain the presence of common host generalist *C. jejuni* lineages.

Pathogen exposure becomes a major factor for zoonoses where zoonotic pathogens can transition between hosts. Our analysis indicates that chicken domestication increased *C. jejuni* host transition at least 15-fold relative to predomestication levels. However, the major driver is intensive poultry production, which has coincided with a more than 100-fold increase in host switching (Fig. 3*C*). Elevated cross-species transmission is an intuitive finding given the vast chicken populations and global dissemination but there is another, possibly more profound impact on zoonotic pathogen evolution. As host populations grow, so do pathogen populations, and this influences the rate of host adaptation (69).

The rapidly expanding chicken gut niche has led to a 50 to 200-fold increase in the *C. jejuni* effective population size (Ne) for many chicken associated strains. This far outpaces the sevenfold growth of the chicken population over the same period (Fig. 3*A*). This disparity suggests that pathogen populations are not only scaling with host population abundance but experiencing ecological shifts that amplify genetic diversity. The scale of Ne expansion may reflect changes such as more efficient transmission, reduced bottlenecks, and greater opportunities for genetic exchange via horizontal gene transfer in the chicken niche (Fig. 1*G*). In bacteria, asexual reproduction means that beneficial mutations compete in different lineages slowing overall population adaptation (clonal interference). However, *C. jejuni* is highly recombinogenic, meaning that clonal interference may be less important and the population adaptive rate may scale with Ne, rather than ln(Ne) as in strictly clonal organisms (70, 71). These conditions can accelerate the generation of genetic diversity and adaptation, enabling strains to respond rapidly to selective pressures in the poultry niche. While our density-dependent transmission simulations do not explicitly model host adaptation, even pathogens that are poor at infecting a host will proliferate in very large, closely packed host populations, such as those observed in modern intensive poultry production systems.

Quantitative Ne estimates are useful for understanding population change and pathogen adaptive potential but do not provide information about the nature of the evolutionary events that enable zoonotic transitions. Wild birds and farmed chickens represent very different niches to the colonizing *C. jejuni*. This not only includes host specific variation in anatomy, physiology, and metabolism but also clear differences in ecology—including stocking density, diet, and dispersal (31). Coupled with the knowledge that some strains have successfully transitioned across this host barrier (31), this allows us to investigate the evolution of zoonoses based on a simple hypothesis. Specifically, when a strain enters a new niche it adapts, and the genetic changes that underlie the most advantageous phenotype adaptations will increase in the population. This basic premise was the principle behind early bacterial GWAS that identified host associated genes and alleles in *Campylobacter* (72). Consistent with this, chicken-adaptive genetic variation occurred in genes involved in diverse functions. For example, the associated elements linked to chicken colonization occurred in genes linked to oxidative stress response, cellular protection, tetracycline resistance, metal homeostasis, and chemotaxis (Fig. 2). These traits likely facilitate proliferation in the new host by optimizing nutrient acquisition and spatial positioning, rather than through metabolic switching, which may play a more limited role in adaptation within poultry. Additionally, some of these traits, including oxidative stress responses, can facilitate food chain transmission (73, 74) and tetracycline resistance may inadvertently increase their ability to persist in humans.

More than half of the chicken-associated loci were linked to oxidative stress response, protection, or sensing. *C. jejuni* is microaerophilic largely because it relies on oxidant-sensitive Fe–S cluster enzymes in central metabolism (75). The hemerythrins HerA and HerB may enhance aerotolerance by protecting these Fe–S enzymes from oxidative damage (75). Mutants lacking *dsbI*, which encodes a periplasmic disulfide bond formation protein, show increased oxidative stress sensitivity compared with other *dsb* mutants, indicating a specific role in redox homeostasis (76). Together, these findings suggest that specialized oxidative stress defenses are key adaptations for survival in the chicken gut or during transmission outside the host.

The role of cytochrome *c* peroxidase (CCP) encoded by *cj0020c* (*docA/ccpA1*) is more complex. Although *C. jejuni* possesses two CCPs (the second encoded by *cj0358*/*ccpA2*), deletion of either gene does not markedly increase sensitivity to external oxidants (77, 78). Instead, these enzymes may use periplasmic hydrogen peroxide as an electron acceptor for energy conservation (79). Notably, *docA* is essential for chick colonization, with mutants showing a 10^5^ fold reduction in colony-forming units of *C. jejuni* in the cecum (78). The *cj0020c* gene overlaps with and is translationally coupled to *cj0019c/docB*, encoding the chemotaxis protein Tlp10 (45); mutants in *cj0019c* also show reduced chick colonization (45). This arrangement suggests DocA might interact with Tlp10, perhaps to signal peroxide availability.

The MerR family of transcription factors is associated with regulation of genes affecting redox state, metal homeostasis, and multidrug resistance (80, 81). These functions are known to be important for *Campylobacter* survival and adaptation, although direct experimental validation in this species is limited. Cj1563, flagged in the GWAS, is most likely to be involved in metal homeostasis; Cys135 and His139 in the C-terminal sensing domain likely ligate a metal ion, perhaps along with Cys72 in the DNA-binding domain. A role in copper homeostasis was previously suggested (42) and this would also be important for oxidative stress management, given the potential for metal-ion catalyzed ROS formation.

It is unclear whether these adaptations primarily confer protection in the chicken host, during environmental transmission, or in both cases. Importantly, however, genes linked to oxidative stress and aerotolerance suggest that association with chicken hosts may increase oxygen tolerance and thereby inadvertently select for traits that would enhance bacterial persistence on food and promote transmission to humans (82). As such, the chicken host environment may drive the evolution of strains that are more likely to cause human infections.

In conclusion, intensive livestock production has done more than amplify pathogen abundance, it has reshaped the evolutionary landscape in which zoonotic bacteria emerge. By creating dense, globally connected host populations, industrial farming increases spillover opportunities, accelerates host switching, and expands pathogen effective population sizes; enhancing adaptive potential. In this context, traits selected for success in livestock, such as stress tolerance and transmission efficiency, can inadvertently promote human infection. More broadly, anthropogenic ecosystem change can generate powerful evolutionary feedback loops, transforming local microbes into globally disseminated zoonotic threats and fundamentally altering the trajectory of pathogen emergence.

## Methods

### Isolate Genomes.

We analyzed genomes of 2,747 isolates (1,892 chicken, 855 wild bird), spanning 1,081 STs and 27 countries from 1979 to 2024 (Dataset S1) from previously published studies (72, 83, 84). Genome quality was assessed with QUAST (85) and PubMLST (39), isolates meeting the following criteria were used in this study: N95 > 800 bases, genome size 1.3 to 2.1 Mb, contig number < 500, and no multiple allele hits in the cgMLST. These are the same as previously reported relaxed thresholds for *C. jejuni* (40), ensuring that as many isolates as possible are included from undersampled wild bird hosts. In our dataset, the genome assembly statistics of assembly size, contig number, length of the largest contig and average contig length were similar in both wild birds and chickens (*SI Appendix*, Fig. S3). Genomes were checked for completeness and contamination using checkM (v1.2.3) (86) and those meeting inclusion criteria were randomly subsampled, so no ST contributed more than five isolates per source. This was performed to reduce the sampling bias, as chickens have considerably more isolate data, while preserving the phylogenetic diversity of the data. All genomes and metadata are available on the pubMLST database (Dataset S1).

### Host Associations and Isolate Diversity.

To allow comparison to established nomenclatures and understand lineage segregation on the phylogenetic tree isolate CC were defined based on sharing of four or more alleles across the seven multilocus sequence typing loci^13^ for initial analysis of CC infecting different host species. Isolates from low frequency sources were removed to give a dataset comprising *C*. *jejuni* genomes from 52,075 human, 41,875 chicken, 10,370 cattle, 3,476 pig, 1,801 turkey, 1,775 goat + sheep, 1,537 wild bird, 598 duck, and 240 goose samples with isolates obtained from both fecal samples and meat. For this analysis, duck and goose isolates came from both wild and domesticated sources. Isolates belonged to 45 CC with varying degrees of host specificity. Heatmaps were generated with pheatmap (v4.4.3) (87) after log transformation and z-score normalization by row to reduce the effect of sampling bias in the different hosts and to emphasize clonal complex segregation by different species. Principal component analysis was performed on log-transformed data and clustered using Ward’s hierarchical algorithm (R stats v4.4.1). We also investigated the fraction of CC composed of chicken and wild bird isolates (Fig. 1*D*). Clonal diversity across wild birds, chickens, humans, and cattle was assessed with iNEXT (v3.0.1) (88), which generated rarefaction curves of ST richness against sample number.

### Phylogenetic Reconstruction and Analysis.

To generate phylogenies, we used Bakta (v1.10.4) for genome annotation, followed by core genome identification and alignment with Panaroo (v1.5.2) (89). The distribution of these core and accessory genes was plotted across the phylogenetic tree (*SI Appendix*, Fig. S2) and prevalence of each gene across wild birds and chickens (Fig. 1*F*). The core genome alignment was used to generate a maximum likelihood phylogenetic tree using RAxML (version 8.2.12) (90) with the GTR + Gamma substitution model and 100 bootstraps. Subsequently, inferred recombined sequence was removed from the alignment using ClonalFrameML (v1.12) (91) as this can obscure clonal relationships, and the trees were visualized using ggtree (v3.14.0).

To compare the amount of recombination between host-associated populations, we calculated patristic distances from the RAxML phylogeny before and after ClonalFrameML adjustment. The proportion of genetic variability attributable to recombination versus mutation for each wild bird and chicken isolates was estimated as

$$Recombinational\;distance\;fraction=\frac{\sum (RAxML\;distance-CFML\;distance)}{\sum RAxML\;distance}.$$

As these tree branches have units of mutations per site, the proportion of genetic distance attributed to recombination will be affected by both length of recombination events, and the number of recombination events, as these events have a minimum length.

Host transition rates on the phylogenetic tree were inferred using TreeBreaker (v1.1) (92) to estimate per branch host transition event probabilities. First, the tree was rooted using the initRoot function in BactDating (93) with 100 iterations, to identify the most probable root location based on tip dates. Then we dated the phylogeny using BactDating run with default settings with 500,000 burn-in iterations, followed by 500,000 iterations sampled every 1,000 steps. Confidence in the dated tree was assessed by plotting root to tip distance against sampling date and confirming coalescence of posterior probability, likelihood, clock rate, coalescent time unit, relaxation parameter and date of root (*SI Appendix*, Figs. S4 and S5). The dated tree was analyzed to estimate per branch host transition probabilities. These probabilities were shared linearly across the branch length and plotted against time. The gradient of this plot was taken between important periods in chicken domestication and farming intensification, and the rate of host transitions was calculated relative to the predomestication rates. While other studies have found similar root dates (94), such estimates in *Campylobacter* species are challenging due to the high recombination rates and limited temporal signal in contemporary datasets (95), under these conditions tools such as bactdating can struggle to accurately date species trees. Consequently, it is probable our tree underestimates the root date, leading to underestimates of fold changes in host transition rates. Additionally, the relatively low numbers of wild bird samples may cause an underestimate in transition rates. For this reason, we also present relative transition rates in the text compared to predomestication rates.

### Bacterial GWAS.

GWAS have been used extensively to understand adaptation in *Campylobacter* (72, 74, 96). To analyze the gene and k-mer associations between hosts we used the Pyseer (v1.3.12) (97) mixed effect model; a phylogeny-aware GWAS tool. These analyses required i) a phylogenetic tree, ii) isolate metadata, and iii) gene or k-mer presence matrices. The phylogeny was generated as described above, and host metadata were curated from PubMLST. Gene presence/absence matrices were obtained with Panaroo with the merging paralog function enabled. Core genome was defined as presence in ≥0.95 of isolates and a gene sequence match ≥0.98 identity. For the k-mer analyses, unitig-caller (v1.3.1) (98) was used to generate unitig (variable length k-mer) profiles. In both cases we generated a genetic relatedness matrix from the phylogenetic tree, this was subsequently used to adjust *P*-values to reduce lineage effects.

Default pyseer filtering removed unitigs and genes present at minor allele frequencies less than 1%. For the gene GWAS, a total of 7,075 genes were identified in the *C. jejuni* pangenome with panaroo. Of these, 5,009 were filtered out of the analysis due to minor allele frequencies of less than 1%, leaving 2,066 variants to test. Similarly, 2,197,848 unitig variants were generated by unitig caller, 890,347 were removed in preprocessing and then 1,307,501 unitigs were tested using the Pyseer. To determine genes associated with k-mer hits, we used the annotation script from Pyseer to map the k-mers back to annotated genomes. These genes were then filtered to give only genes associated with ≥ 10 significant k-mers.

For both gene and k-mer analyses, significance thresholds were set using Bonferroni correction at α = 0.05. MAF or gene frequency had to be >5% and <95%, with positive beta values, to indicate chicken association. Results were plotted against the phylogenetic tree, so the distribution of genes and kmers could be compared to host partitioning (Fig. 2*C*). Additionally, for the k-mer analysis, Manhattan plots were created and the k-mer associated genes were plotted with MAF and max *P*-value. As additional quality controls we checked the average contig length, average assembly length, and number of contigs between isolates in the two host types, these showed similar distributions (*SI Appendix*, Fig. S3). Narrow sense heritability (h^2^) for host association was also calculated in Pyseer using the genetic relatedness matrix.

### Effective Population Sizes.

The changes in effective population size (Ne) over 63 y (dates 1960–2023) was estimated for seven chicken-associated CC, each represented by at least 100 genomes meeting required quality thresholds (Dataset S2). CC with more than 300 isolates were randomly subsampled to give 300. Core genome alignments were generated with MAFFT (99) using default parameters in Genome Comparator (PubMLST) and *Campylobacter* version 2 core genome MLST, and maximum-likelihood phylogenies were inferred with FastTree (v2.1.11) (100). Phylogenies were dated with BactDating (v1.1.2) (93), using initRoot with 100 iterations for rooting and 100,000 MCMC iterations for dating. Confidence in the dated tree was accessed by plotting root to tip distance against sampling date and ensuring *R^2^* > 0.1 and confirming coalescence of posterior probability, likelihood, clock rate, coalescent time unit, relaxation parameter and date of root. Effective population size trajectories were then inferred with Skygrowth (v0.3.1) (101) using 100 intervals (res = 100) and the smoothing parameter set at tau0 = 10.

### Modeling.

To investigate the dynamics of *Campylobacter* transition as host population sizes changes, we used an ordinary differential equation model. The model simulates the infection dynamics of *C. jejuni* strains in two linked host populations, representing chickens and wild birds. Eqs. **1**–**4** give the rate of change of the numbers of susceptible (*S_i_*), infected with strains 1 or 2 (*I_i1_* and *I_i2_*), or infected with both strains (*I_i1,2_*) hosts of species *i*. We made the simplifying assumption that there is no memory immune response to *C. jejuni*, so upon recovery, hosts return to being susceptible and can be reinfected. For broiler chickens, this is plausible because birds are often harvested before immune maturation is complete (102) and maternally inherited antibodies decline rapidly in the first 2 wk of age (103). In wild birds, immune responses are less well understood and asymptomatic carriage may frequently occur (25). The total population size of host *i (N_i_)* is kept constant by making the birth rate equal to the mortality rate (*μ_i_*). 1/*μ_i_* gives the average lifespan of host species *i*. We assumed transmission of *C. jejuni* to be density dependent, meaning that the force of infection for strain 1 for host species *i* ($\lambda_{i,1}$) or strain 2 for host species *i* ($\lambda_{i,2}$) is a function of the number of hosts currently infected with that strain (Eqs. **6** and **7**).

To define the forces of infection we additionally used a contact rate scaling parameter (*c_ij_*), representing how the number of contacts a host of type *i* has with feces from host type *j* per unit time of the model scales with the overall number of host of type *j.* We combined the contact rate scaling parameter with a probability of infection per contact representing the probability that a host of type *i* becomes infected with strain 1 (*p_i1_*) or strain 2 (*p_i2_*) if it has contact with the feces of a host infected with the relevant strain. To investigate the potential impact of host specificity of strains, we introduced parameter *k* and made p_12_ = p_11_/k (i.e., the probability of host type 1 being infected with strain 2 is k times less than the probability of host type 1 being infected with strain 1). Similarly, p_21_ = p_22_/k. Finally, we allowed for the possibility of interference between the strains, by introducing parameter *d*. Cases of coinfection with both strains (*I_i1,2_*) shed each strain at (1-*d*) times the rate of a singly infected bird.

Rate equations:

[1] $$\frac{dS_{i}}{\mathrm{dt}}=\mu_{i}N_{i}+\sigma (I_{i,1}+I_{i,2})-(\lambda_{i,1}+\lambda_{i,2}+\mu_{i})S_{i},$$

[2] $$\frac{dI_{i,1}}{\mathrm{dt}}=\lambda_{i,1}S_{i}+\sigma I_{i,1,2}-\left(\sigma +\lambda_{i,2}+\mu_{i}\right)I_{i,1},$$

[3] $$\frac{dI_{i,2}}{\mathrm{dt}}=\lambda_{i,2}S_{i}+\sigma I_{i,1,2}-(\sigma +\lambda_{i,1}+\mu_{i})I_{i,2},$$

[4] $$\frac{dI_{i,1,2}}{\mathrm{dt}}=\lambda_{i,1}I_{i,2}+\lambda_{i,2}I_{i,1}-(2\sigma +\mu_{i})I_{i,1,2}.$$

Total population:

[5] $$N_{i}=S_{i}+I_{i,1}+I_{i,2}+I_{i,1,2}.$$

Force of infection:

[6] $$\lambda_{i,1}=p_{i,1}(c_{i,i}(I_{i,1}+0.5I_{i,1,2})+c_{i,j}(I_{j,1}+(1-d)I_{j,1,2})),$$

[7] $$\lambda_{i,2}=p_{i,2}(c_{i,i}(I_{i,2}+0.5I_{i,1,2})+c_{i,j}(I_{j,2}+(1-d)I_{j,1,2})).$$

The ode15s solver in Matlab version 2025a was used to find numerical solutions to this system. We then explored the circumstances in the model under which *C. jejuni* strains with a specialism to either host type 1 or host type 2 stay within, or spread between, the two host populations, as we varied the size of one population (*N_1_*) relative to the other (*N_2_*). Parameter values used in individual simulations are given in the modeling figures and their legends. It should be noted that it is challenging to estimate the true values of many of the parameters of this model, and our simulations are intended as an informative abstraction of reality, rather than to make a prediction about a specific population.

## Supplementary Material

Appendix 01 (PDF)

Dataset S01 (XLSX)

Dataset S02 (XLSX)

## Data Availability

Datasets analysed during the current study data have been deposited in FigShare (https://doi.org/10.6084/m9.figshare.31410435 (104)). All other data are included in the manuscript and/or supporting information.

## References

[1] C. N. Waters , The anthropocene is functionally and stratigraphically distinct from the holocene. Science **351**, aad2622-10 (2016). 26744408 10.1126/science.aad2622

[2] Y. M. Bar-On, R. Phillips, R. Milo, The biomass distribution on Earth. Proc. Natl. Acad. Sci. U.S.A. **115**, 6506–6511 (2018). 29784790 10.1073/pnas.1711842115 PMC6016768

[3] C. Linnaeus, Systema Naturae (Laurentii Salvii, ed. 10, 1758).

[4] C. E. Bennett , The broiler chicken as a signal of a human reconfigured biosphere. R. Soc. Open Sci. **5**, 180325 (2018). 30662712 10.1098/rsos.180325 PMC6304135

[5] Food and Agriculture Organization for the United Nations, FAOSTAT: Crops and livestock products. FAOSTAT. https://www.fao.org/faostat/en/#data/QCL. Accessed 23 July 2025.

[6] J. A. Robbins, M. A. G. von Keyserlingk, D. Fraser, D. M. Weary, Farm Size and Animal Welfare (American Society of Animal Science, 2016). 10.2527/jas.2016-0805 28046157

[7] B. B. Oakley , The poultry-associated microbiome: Network analysis and farm-to-fork characterizations. PLoS One **8**, e57190 (2013). 23468931 10.1371/journal.pone.0057190 PMC3584146

[8] P. Antunes, J. Mourão, J. Campos, L. Peixe, Salmonellosis: The role of poultry meat. Clin. Microbiol. Infect. **22**, 110–121 (2016). 26708671 10.1016/j.cmi.2015.12.004

[9] A. C. Fluit, Livestock-associated *Staphylococcus aureus*. Clin. Microbiol. Infect. **18**, 735–744 (2012). 22512702 10.1111/j.1469-0691.2012.03846.x

[10] A. Singh Dhillon , *Campylobacter jejuni* infection, in broiler chickens. Avian Dis. **50**, 55–58 (2006). 16617982 10.1637/7411-071405R.1

[11] C. K. Smith , *Campylobacter* colonization of the chicken induces a proinflammatory response in mucosal tissues. FEMS Immunol. Med. Microbiol. **54**, 114–121 (2008). 18647351 10.1111/j.1574-695X.2008.00458.x

[12] F. J. Gormley , *Campylobacter* colonization and proliferation in the broiler chicken upon natural field challenge is not affected by the bird growth rate or breed. Appl. Environ. Microbiol. **80**, 6733–6738 (2014). 25172857 10.1128/AEM.02162-14 PMC4249045

[13] S. K. Sheppard , *Campylobacter* genotyping to determine the source of human infection. Clin. Infect. Dis. **48**, 1072–1078 (2009). 19275496 10.1086/597402 PMC3988352

[14] N. Arning, S. K. Sheppard, S. Bayliss, D. A. Clifton, D. J. Wilson, Machine learning to predict the source of campylobacteriosis using whole genome data. PLoS Genet. **17**, e1009436 (2021). 34662334 10.1371/journal.pgen.1009436 PMC8553134

[15] Q. Jehanne , Genome-wide identification of host-segregating single-nucleotide polymorphisms for source attribution of clinical *Campylobacter* coli Isolates. Appl. Environ. Microbiol. **86**, e01787-20 (2020). 33036986 10.1128/AEM.01787-20 PMC7688228

[16] E. Berthenet , Source attribution of *Campylobacter jejuni* shows variable importance of chicken and ruminants reservoirs in non-invasive and invasive French clinical isolates. Sci. Rep. **9**, 8098 (2019). 31147581 10.1038/s41598-019-44454-2 PMC6542803

[17] A. Thépault , Genome-wide identification of host-segregating epidemiological markers for source attribution in *Campylobacter* *jejuni*. Appl. Environ. Microbiol. **83**, e03085-16 (2017). 28115376 10.1128/AEM.03085-16 PMC5359498

[18] A. J. Cody, M. C. J. Maiden, N. J. C. Strachan, N. D. McCarthy, A systematic review of source attribution of human campylobacteriosis using multilocus sequence typing. Eurosurveillance **24**,1800696 (2019). 31662159 10.2807/1560-7917.ES.2019.24.43.1800696 PMC6820127

[19] B. Pascoe , Machine learning to attribute the source of *Campylobacter* infections in the United States: A retrospective analysis of national surveillance data. J. Infect. **89**, 106265 (2024). 39245152 10.1016/j.jinf.2024.106265 PMC7617841

[20] I. D. Ogden , *Campylobacter* excreted into the environment by animal sources: Prevalence, concentration shed, and host association. Foodborne Pathog. Dis. **6**, 1161–1170 (2009). 19839759 10.1089/fpd.2009.0327 PMC3985071

[21] E. Mourkas , Gene pool transmission of multidrug resistance among *Campylobacter* from livestock, sewage and human disease. Environ. Microbiol. **21**, 4597–4613 (2019). 31385413 10.1111/1462-2920.14760 PMC6916351

[22] E. Mourkas , Agricultural intensification and the evolution of host specialism in the enteric pathogen *Campylobacter jejuni*. Proc. Natl. Acad. Sci. U.S.A. **117**, 11018–11028 (2020). 32366649 10.1073/pnas.1917168117 PMC7245135

[23] N. O. Kaakoush, N. Castaño-Rodríguez, H. M. Mitchell, S. M. Man, Global epidemiology of *Campylobacter* infection. Clin. Microbiol. Rev. **28**, 687–720 (2015). 26062576 10.1128/CMR.00006-15 PMC4462680

[24] C. Atterby , The potential of isolation source to predict colonization in avian hosts: A case study in *Campylobacter jejuni* strains from three bird species. Front. Microbiol. **9**, 591 (2018). 29651281 10.3389/fmicb.2018.00591 PMC5884941

[25] P. Griekspoor , Marked host specificity and lack of phylogeographic population structure of *Campylobacter jejuni* in wild birds. Mol. Ecol. **22**, 1463–1472 (2013). 23356487 10.1111/mec.12144 PMC3596980

[26] A. J. H. Leatherbarrow , *Campylobacter lari*: Genotype and antibiotic resistance of isolates from cattle, wildlife and water in an area of mixed dairy farmland in the United Kingdom. Environ. Microbiol. **9**, 1772–1779 (2007). 17564610 10.1111/j.1462-2920.2007.01295.x

[27] S. K. Sheppard , Niche segregation and genetic structure of *Campylobacter jejuni* populations from wild and agricultural host species. Mol. Ecol. **20**, 3484–3490 (2011). 21762392 10.1111/j.1365-294X.2011.05179.x PMC3985062

[28] N. P. French , Molecular epidemiology of *Campylobacter jejuni* isolates from wild-bird fecal material in children’s playgrounds. Appl. Environ. Microbiol. **75**, 779–783 (2009). 19047378 10.1128/AEM.01979-08 PMC2632120

[29] F. M. Colles, K. E. Dingle, A. J. Cody, M. C. J. Maiden, Comparison of *Campylobacter* populations in wild geese with those in starlings and free-range poultry on the same farm. Appl. Environ. Microbiol. **74**, 3583–3590 (2008). 18390684 10.1128/AEM.02491-07 PMC2423018

[30] F. M. Colles , *Campylobacter* infection of broiler chickens in a free-range environment. Environ. Microbiol. **10**, 2042–2050 (2008). 18412548 10.1111/j.1462-2920.2008.01623.x PMC2702501

[31] E. Mourkas , Host ecology regulates interspecies recombination in bacteria of the genus *Campylobacter*. Elife **11**, e73552 (2022). 35191377 10.7554/eLife.73552 PMC8912921

[32] S. K. Sheppard , Cryptic ecology among host generalist *Campylobacter jejuni* in domestic animals. Mol. Ecol. **23**, 2442–2451 (2014). 24689900 10.1111/mec.12742 PMC4237157

[33] F. M. Colles, N. D. McCarthy, C. M. Bliss, R. Layton, M. C. J. Maiden, The long-term dynamics of *Campylobacter* colonizing a free-range broiler breeder flock: An observational study. Environ. Microbiol. **17**, 938–946 (2015). 25588789 10.1111/1462-2920.12415 PMC4390391

[34] B. A. Jones , Zoonosis emergence linked to agricultural intensification and environmental change. Proc. Natl. Acad. Sci. U.S.A. **110**, 8399–8404 (2013). 23671097 10.1073/pnas.1208059110 PMC3666729

[35] S. K. Sheppard, D. S. Guttman, J. R. Fitzgerald, Population genomics of bacterial host adaptation. Nat. Rev. Genet. **19**, 549–565 (2018). 29973680 10.1038/s41576-018-0032-z

[36] B. L. Dearlove , Rapid host switching in generalist *Campylobacter* strains erodes the signal for tracing human infections. ISME J. **10**, 721–729 (2016). 26305157 10.1038/ismej.2015.149 PMC4677457

[37] D. J. Woodcock , Genomic plasticity and rapid host switching can promote the evolution of generalism: A case study in the zoonotic pathogen *Campylobacter*. Sci. Rep. **7**, 9650 (2017). 28851932 10.1038/s41598-017-09483-9 PMC5575054

[38] World Health Organization, Campylobacter. (2020). https://www.who.int/news-room/fact-sheets/detail/campylobacter. Accessed 15 September 2025.

[39] K. A. Jolley, J. E. Bray, M. C. J. Maiden, Open-access bacterial population genomics: BIGSdb software, the PubMLST.org website and their applications. Wellcome Open Res. **3**, 124 (2018). 30345391 10.12688/wellcomeopenres.14826.1 PMC6192448

[40] E. Mourkas , Proximity to humans is associated with antimicrobial-resistant enteric pathogens in wild bird microbiomes. Curr. Biol. **34**, 3955–3965.e4 (2024). 39142288 10.1016/j.cub.2024.07.059

[41] L. Avrain, C. Vernozy-Rozand, I. Kempf, Evidence for natural horizontal transfer of tetO gene between *Campylobacter jejuni* strains in chickens. J. Appl. Microbiol. **97**, 134–140 (2004). 15186450 10.1111/j.1365-2672.2004.02306.x

[42] S. J. Hall, A. Hitchcock, C. S. Butler, D. J. Kelly, A multicopper oxidase (Cj1516) and a CopA homologue (Cj1161) are major components of the copper homeostasis system of *Campylobacter jejuni*. J. Bacteriol. **190**, 8075–8085 (2008). 18931123 10.1128/JB.00821-08 PMC2593206

[43] M. Cerasi, S. Ammendola, A. Battistoni, Competition for zinc binding in the host-pathogen interaction. Front. Cell. Infect. Microbiol. **3**, 108 (2013). 24400228 10.3389/fcimb.2013.00108 PMC3872050

[44] L. M. Davis, T. Kakuda, V. J. DiRita, A *Campylobacter jejuni* znuA orthologue is essential for growth in low-zinc environments and chick colonization. J. Bacteriol. **191**, 1631–1640 (2009). 19103921 10.1128/JB.01394-08 PMC2648198

[45] D. R. Hendrixson, V. J. DiRita, Identification of *Campylobacter jejuni* genes involved in commensal colonization of the chick gastrointestinal tract. Mol. Microbiol. **52**, 471–484 (2004). 15066034 10.1111/j.1365-2958.2004.03988.x

[46] K. T. Elliott, V. J. DiRita, Characterization of CetA and CetB, a bipartite energy taxis system in *Campylobacter jejuni*. Mol. Microbiol. **69**, 1091–1103 (2008). 18631239 10.1111/j.1365-2958.2008.06357.x PMC2628428

[47] M. R. Yen , The ubiquitous ThrE family of putative transmembrane amino acid efflux transporters. Res. Microbiol. **153**, 19–25 (2002). 11881894 10.1016/s0923-2508(01)01281-5

[48] N. Garg , Genes Linking copper trafficking and homeostasis to the biogenesis and activity of the cbb3-type cytochrome c oxidase in the enteric pathogen *Campylobacter jejuni*. Front. Microbiol. **12**, 683260 (2021). 34248902 10.3389/fmicb.2021.683260 PMC8267372

[49] J. Velayudhan, M. A. Jones, P. A. Barrow, D. J. Kelly, L-serine catabolism via an oxygen-labile L-serine dehydratase is essential for colonization of the avian gut by *Campylobacter jejuni*. Infect. Immun. **72**, 260–268 (2004). 14688104 10.1128/IAI.72.1.260-268.2004 PMC343963

[50] J. J. Rasmussen , *Campylobacter jejuni* carbon starvation protein A (CstA) is involved in peptide utilization, motility and agglutination, and has a role in stimulation of dendritic cells. J. Med. Microbiol. **62**, 1135–1143 (2013). 23682166 10.1099/jmm.0.059345-0

[51] M. S. Wang , 863 genomes reveal the origin and domestication of chicken. Cell Res. **30**, 693–701 (2020). 32581344 10.1038/s41422-020-0349-y PMC7395088

[52] J. Peters , The biocultural origins and dispersal of domestic chickens. Proc. Natl. Acad. Sci. U.S.A. **119**, e2121978119 (2022). 35666876 10.1073/pnas.2121978119 PMC9214543

[53] G. Méric , Lineage-specific plasmid acquisition and the evolution of specialized pathogens in Bacillus thuringiensis and the Bacillus cereus group. Mol. Ecol. **27**, 1524–1540 (2018). 29509989 10.1111/mec.14546 PMC5947300

[54] G. Méric , Convergent amino acid signatures in polyphyletic *Campylobacter jejuni* subpopulations suggest human niche tropism. Genome Biol. Evol. **10**, 763–774 (2018). 29452359 10.1093/gbe/evy026 PMC5841378

[55] W. Monteith , Everything is everywhere but *Escherichia coli* adapts to different niches. ISME J. **20**, wraf267 (2026). 41408707 10.1093/ismejo/wraf267 PMC12815263

[56] M. E. J. Woolhouse, D. T. Haydon, R. Antia, Emerging pathogens: The epidemiology and evolution of species jumps. Trends Ecol. Evol. **20**, 238–244 (2005). 16701375 10.1016/j.tree.2005.02.009 PMC7119200

[57] A. J. McMichael, Environmental and social influences on emerging infectious diseases: Past, present and future. Philos. Trans. R. Soc. B Biol. Sci. **359**, 1049–1058 (2004). 10.1098/rstb.2004.1480 PMC1693387 15306389

[58] D. G. Newell, C. Fearnley, Sources of *Campylobacter* colonization in broiler chickens. Appl. Environ. Microbiol. **69**, 4343–4351 (2003). 12902214 10.1128/AEM.69.8.4343-4351.2003 PMC169125

[59] Innovations Report, Chicken run !–on the environmental impact of broiler production systems–Innovations Report. (2005). https://www.innovations-report.com/agriculture-environment/agricultural-and-forestry-science/report-48133/. Accessed 15 September 2025.

[60] S. Yano , Intestinal carriage and excretion of *Campylobacter jejuni* in chickens exposed at different ages. J. Food Prot. **77**, 1184–1187 (2014). 24988026 10.4315/0362-028X.JFP-14-061

[61] S. K. Sheppard , *Campylobacter* genotypes from food animals, environmental sources and clinical disease in Scotland 2005/6. Int. J. Food Microbiol. **134**, 96–103 (2009). 19269051 10.1016/j.ijfoodmicro.2009.02.010 PMC3985063

[62] J. Waldenström , Prevalence of *Campylobacter jejuni*, *Campylobacter lari*, and *Campylobacter coli* in different ecological guilds and taxa of migrating birds. Appl. Environ. Microbiol. **68**, 5911–5917 (2002). 12450810 10.1128/AEM.68.12.5911-5917.2002 PMC134389

[63] G. Kapperud, O. Rosef, Avian wildlife reservoir of *Campylobacter* fetus subsp. jejuni, Yersinia spp., and Salmonella spp. in Norway. Appl. Environ. Microbiol. **45**, 375–380 (1983). 6338824 10.1128/aem.45.2.375-380.1983 PMC242295

[64] T. Broman , *Campylobacter jejuni* in black-headed gulls (*Larus ridibundus*): Prevalence, genotypes, and influence on *C. jejuni* epidemiology. J. Clin. Microbiol. **40**, 4594–4602 (2002). 12454158 10.1128/JCM.40.12.4594-4602.2002 PMC154640

[65] A. D. Pearson , Colonization of broiler chickens by waterborne *Campylobacter jejuni*. Appl. Environ. Microbiol. **59**, 987–996 (1993). 8476300 10.1128/aem.59.4.987-996.1993 PMC202227

[66] A. M. Weis , Genomic comparison of *Campylobacter* spp. and their potential for zoonotic transmission between birds, primates, and livestock. Appl. Environ. Microbiol. **82**, 7165–7175 (2016). 27736787 10.1128/AEM.01746-16 PMC5118927

[67] F. M. Colles , Dynamics of *Campylobacter* colonization of a natural host, Sturnus vulgaris (European Starling). Environ. Microbiol. **11**, 258–267 (2009). 18826435 10.1111/j.1462-2920.2008.01773.x PMC2702492

[68] N. J. Stern, J. S. Bailey, L. C. Blankenship, N. A. Cox, F. Mchan, Colonization characteristics of *Campylobacter jejuni* in chick ceca. Am. Assoc. Avian Pathol. **32**, 330–334 (1988). 3401176

[69] A. Papkou, R. Schalkowski, M. C. Barg, S. Koepper, H. Schulenburg, Population size impacts host-pathogen coevolution. Proc. Royal Soc. B Biol. Sci. **288**, 20212269 (2021). 10.1098/rspb.2021.2269 PMC8670963 34905713

[70] M. M. Desai, D. S. Fisher, Beneficial mutation-selection balance and the effect of linkage on positive selection. Genetics **176**, 1759–1798 (2007). 17483432 10.1534/genetics.106.067678 PMC1931526

[71] R. A. Neher, O. Hallatschek, Genealogies of rapidly adapting populations. Proc. Natl. Acad. Sci. U.S.A. **110**, 437–442 (2013). 23269838 10.1073/pnas.1213113110 PMC3545819

[72] S. K. Sheppard , Genome-wide association study identifies vitamin B5 biosynthesis as a host specificity factor in *Campylobacter*. Proc. Natl. Acad. Sci. U.S.A. **110**, 11923–11927 (2013). 23818615 10.1073/pnas.1305559110 PMC3718156

[73] J. K. Calland , Genomic tailoring of autogenous poultry vaccines to reduce *Campylobacter* from farm to fork. NPJ Vaccines **9**, 105 (2024). 38866805 10.1038/s41541-024-00879-z PMC11169640

[74] K. Yahara , Genome-wide association of functional traits linked with *Campylobacter jejuni* survival from farm to fork. Environ. Microbiol. **19**, 361–380 (2017). 27883255 10.1111/1462-2920.13628

[75] J. J. Kendall, A. M. Barrero-Tobon, D. R. Hendrixson, D. J. Kelly, Hemerythrins in the microaerophilic bacterium *Campylobacter jejuni* help protect key iron-sulphur cluster enzymes from oxidative damage. Environ. Microbiol. **16**, 1105–1121 (2014). 24245612 10.1111/1462-2920.12341 PMC4257069

[76] A. M. Banaś , C8J–1298, a bifunctional thiol oxidoreductase of *Campylobacter jejuni*, affects Dsb (disulfide bond) network functioning. PLoS One **15**, e0230366 (2020). 32203539 10.1371/journal.pone.0230366 PMC7089426

[77] E. J. Guccione , Transcriptome and proteome dynamics in chemostat culture reveal how *Campylobacter jejuni* modulates metabolism, stress responses and virulence factors upon changes in oxygen availability. Environ. Microbiol. **19**, 4326–4348 (2017). 28892295 10.1111/1462-2920.13930 PMC5656828

[78] L. K. Bingham-Ramos, D. R. Hendrixson, Characterization of two putative cytochrome c peroxidases of *Campylobacter jejuni* involved in promoting commensal colonization of poultry. Infect. Immun. **76**, 1105–1114 (2008). 18086814 10.1128/IAI.01430-07 PMC2258805

[79] A. J. Taylor, D. J. Kelly, The function, biogenesis and regulation of the electron transport chains in *Campylobacter jejuni*: New insights into the bioenergetics of a major food-borne pathogen. Adv. Microb. Physiol. **74**, 239–329 (2019). 31126532 10.1016/bs.ampbs.2019.02.003

[80] G. Tulin, N. R. Figueroa, S. K. Checa, F. C. Soncini, The multifarious MerR family of transcriptional regulators. Mol. Microbiol. **121**, 230–242 (2024). 38105009 10.1111/mmi.15212

[81] M. A. Pennella, D. P. Giedroc, Structural determinants of metal selectivity in prokaryotic metal-responsive transcriptional regulators. BioMetals **18**, 413–428 (2005). 16158234 10.1007/s10534-005-3716-8

[82] E. Oh, L. McMullen, B. Jeon, High prevalence of hyper-aerotolerant *Campylobacter jejuni* in retail poultry with potential implication in human infection. Front. Microbiol. **6**, 167793 (2015). 10.3389/fmicb.2015.01263 PMC4641907 26617597

[83] A. J. Cody , Real-time genomic epidemiological evaluation of human *Campylobacter* isolates by use of whole-genome multilocus sequence typing. J. Clin. Microbiol. **51**, 2526–2534 (2013). 23698529 10.1128/JCM.00066-13 PMC3719633

[84] K. K. Cooper , Sharing of cmeRABC alleles between *C*. *coli* and *C*. *jejuni* associated with extensive drug resistance in *Campylobacter* isolates from infants and poultry in the Peruvian Amazon. mBio **16**, e02054-24 (2025). 39727415 10.1128/mbio.02054-24 PMC11796421

[85] A. Gurevich, V. Saveliev, N. Vyahhi, G. Tesler, QUAST: Quality assessment tool for genome assemblies. Bioinformatics **29**, 1072–1075 (2013). 23422339 10.1093/bioinformatics/btt086 PMC3624806

[86] D. H. Parks, M. Imelfort, C. T. Skennerton, P. Hugenholtz, G. W. Tyson, CheckM: Assessing the quality of microbial genomes recovered from isolates, single cells, and metagenomes. Genome Res. **25**, 1043–1055 (2015). 25977477 10.1101/gr.186072.114 PMC4484387

[87] R. Kolde, pheatmap: Pretty Heatmaps. https://github.com/raivokolde/pheatmap. Accessed 12 October 2025.

[88] A. Chao , Rarefaction and extrapolation with Hill numbers: A framework for sampling and estimation in species diversity studies. Ecol. Monogr. **84**, 45–67 (2014).

[89] G. Tonkin-Hill , Producing polished prokaryotic pangenomes with the Panaroo pipeline. Genome Biol. **21**, 180 (2020). 32698896 10.1186/s13059-020-02090-4 PMC7376924

[90] A. M. Kozlov, D. Darriba, T. Flouri, B. Morel, A. Stamatakis, RAxML-NG: A fast, scalable and user-friendly tool for maximum likelihood phylogenetic inference. Bioinformatics **35**, 4453–4455 (2019). 31070718 10.1093/bioinformatics/btz305 PMC6821337

[91] X. Didelot, D. J. Wilson, ClonalFrameML: Efficient inference of recombination in whole bacterial genomes. PLoS Comput. Biol. **11**, e1004041 (2015). 25675341 10.1371/journal.pcbi.1004041 PMC4326465

[92] M. Azim Ansari, X. Didelot, Bayesian inference of the evolution of a phenotype distribution on a phylogenetic tree. Genetics **204**, 89–98 (2016). 27412711 10.1534/genetics.116.190496 PMC5012407

[93] X. Didelot, N. J. Croucher, S. D. Bentley, S. R. Harris, D. J. Wilson, Bayesian inference of ancestral dates on bacterial phylogenetic trees. Nucleic Acids Res. **46**, e134 (2018). 30184106 10.1093/nar/gky783 PMC6294524

[94] D. J. Wilson , Rapid evolution and the importance of recombination to the gastroenteric pathogen *Campylobacter jejuni*. Mol. Biol. Evol. **26**, 385–397 (2009). 19008526 10.1093/molbev/msn264 PMC2639114

[95] J. K. Calland , Quantifying bacterial evolution in the wild: A birthday problem for *Campylobacter* lineages. PLoS Genet. **17**, e1009829 (2021). 34582435 10.1371/journal.pgen.1009829 PMC8500405

[96] S. Peters , *Campylobacter jejuni* genotypes are associated with post-infection irritable bowel syndrome in humans. Commun. Biol. **4**, 1015–1032 (2021). 34462533 10.1038/s42003-021-02554-8 PMC8405632

[97] J. A. Lees, M. Galardini, S. D. Bentley, J. N. Weiser, J. Corander, Pyseer: A comprehensive tool for microbial pangenome-wide association studies. Bioinformatics **34**, 4310–4312 (2018). 30535304 10.1093/bioinformatics/bty539 PMC6289128

[98] G. Holley, P. Melsted, Bifrost: Highly parallel construction and indexing of colored and compacted de Bruijn graphs. Genome Biol. **21**, 1–20 (2020). 10.1186/s13059-020-02135-8 PMC7499882 32943081

[99] K. Katoh, D. M. Standley, MAFFT multiple sequence alignment software version 7: Improvements in performance and usability. Mol. Biol. Evol. **30**, 772–780 (2013). 23329690 10.1093/molbev/mst010 PMC3603318

[100] M. N. Price, P. S. Dehal, A. P. Arkin, FastTree 2—Approximately maximum-likelihood trees for large alignments. PLoS One **5**, e9490 (2010). 20224823 10.1371/journal.pone.0009490 PMC2835736

[101] E. M. Volz, X. Didelot, Modeling the growth and decline of pathogen effective population size provides insight into epidemic dynamics and drivers of antimicrobial resistance. Syst. Biol. **67**, 719–728 (2018). 29432602 10.1093/sysbio/syy007 PMC6005154

[102] B. Song , Effects of age on immune function in broiler chickens. J. Anim. Sci. Biotechnol. **12**, 42 (2021). 33731181 10.1186/s40104-021-00559-1 PMC7971956

[103] R. Yosipovich , Overcoming the susceptibility gap between maternal antibody disappearance and auto-antibody production. Vaccine **33**, 472–478 (2015). 25444785 10.1016/j.vaccine.2014.10.043

[104] O. Kyne, B. S. Penman, D. Kelly, S. K. Sheppard, Genomes used in chicken campylobacter jejuni evolution paper. Figshare. 10.6084/m9.figshare.31410435. Deposited 2 March 2026.

