# Supplementary material for: Accelerating Campylobacter zoonosis in the Anthropocene
Source: Proc Natl Acad Sci U S A. 2026 Jul 27;123(32):e2609969123. doi: 10.1073/pnas.2609969123 (PMC13462783; doi:10.1073/pnas.2609969123)
Supplement: Supplementary file 1 [file pnas.2609969123.sapp.pdf]

Supplementary Materials

Supplementary Table 1. GWAS results showing genes with positive beta values (chicken associated) and with more than 10 k-mer hits.

Supplementary Fig. 1. Phylogenetic structure of clonal complexes included in host distribution analyses. Maximum-likelihood phylogeny inferred with RAxML showing the 43 clonal complexes analysed in the host distribution analysis (Fig. 1A,B). Branch colours correspond to the clusters defined in the heatmap and principal component analysis (PCA). The tree indicates that some, but not all, clusters form phylogenetically distinct groupings. These colours directly relate to clusters in the PCA (Figure 1B) as follows: clusters 1 (red), cluster 2 (blue), cluster 3 (green), cluster 4 (purple), cluster 5 (orange).

Supplementary Fig. 2, Fraction of genomes containing tet(O) against isolation date. This shows that in our data there was no *tet(O)* present in our dataset till 2015 and then subsequent years fluctuate between 20% and 45%.

Supplementary Fig. 3. Genome assembly quality control metrics. Distributions of assembly quality metrics, including average contig length (avg_len), longest contig length (max_len), total number of contigs (num_seqs), and total assembly length (sum_len), comparing chicken and wild bird isolates. Similar distributions between host groups indicate that downstream analyses are unlikely to be driven by assembly artifacts.

Supplementary Fig. 4. Pan-genome gene distribution across isolates. Presence–absence patterns of all genes identified by Panaroo across 2,747 chicken and wild bird isolates, plotted alongside the phylogenetic tree. Chicken isolates are shown in orange and wild bird isolates in green. The distribution demonstrates a typical pan-genome structure, with genes either present at low frequency (accessory genome) or conserved across most or all isolates (core genome).

Supplementary Fig. 5. Time-calibrated phylogeny inferred with BactDating. Dated phylogeny of the 2,747 isolates inferred using BactDating, with 95% confidence intervals for node dates. As expected for time-calibrated trees, uncertainty increases toward deeper nodes, particularly near the root (~19,000 BCE).

Supplementary Fig. 6. BactDating MCMC trace diagnostics. Trace plots from the BactDating analysis showing stabilization of key parameters across 1,000 MCMC iterations, including posterior probability, likelihood, prior, root date, clock rate, coalescent time unit, and relaxation parameter, indicating adequate convergence.

gene	Number of kmer hits	-log10(maxp)	Average allele frequency	Average minor allele frequency	Average beta value
**znuA**	22	20.13194	0.621591	0.378409	0.147700
**cetA**	153	17.08884	0.745046	0.254954	0.190641
**dsbI**	42	14.17328	0.782738	0.217262	0.192476
**cj0020c**	19	13.31876	0.705526	0.294474	0.189711
**herB**	11	11.84164	0.664091	0.335909	0.119309
**cj1165c**	21	11.00833	0.581333	0.418667	0.249810

Supplementary Table 1

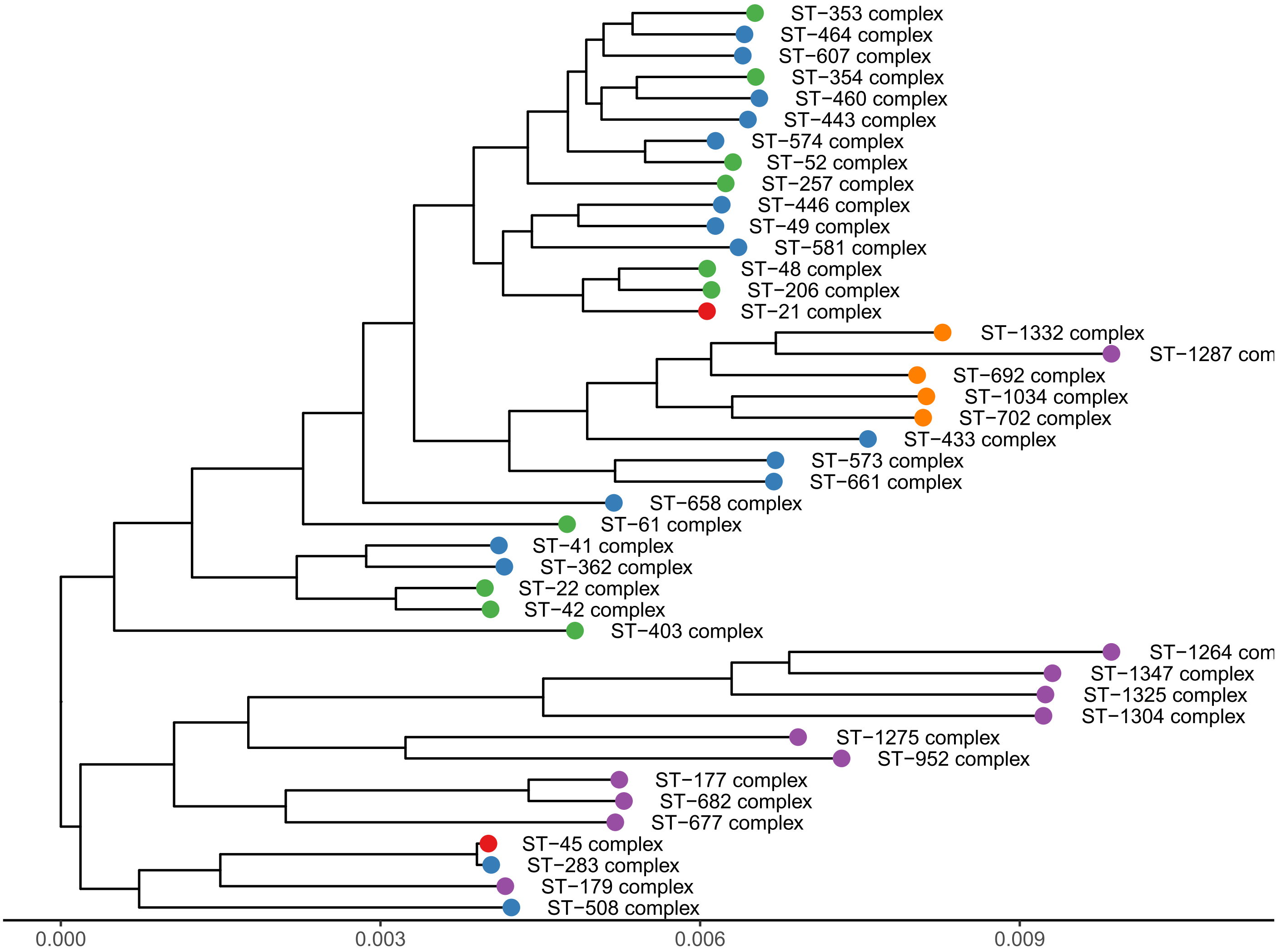

Supplementary Figure 1

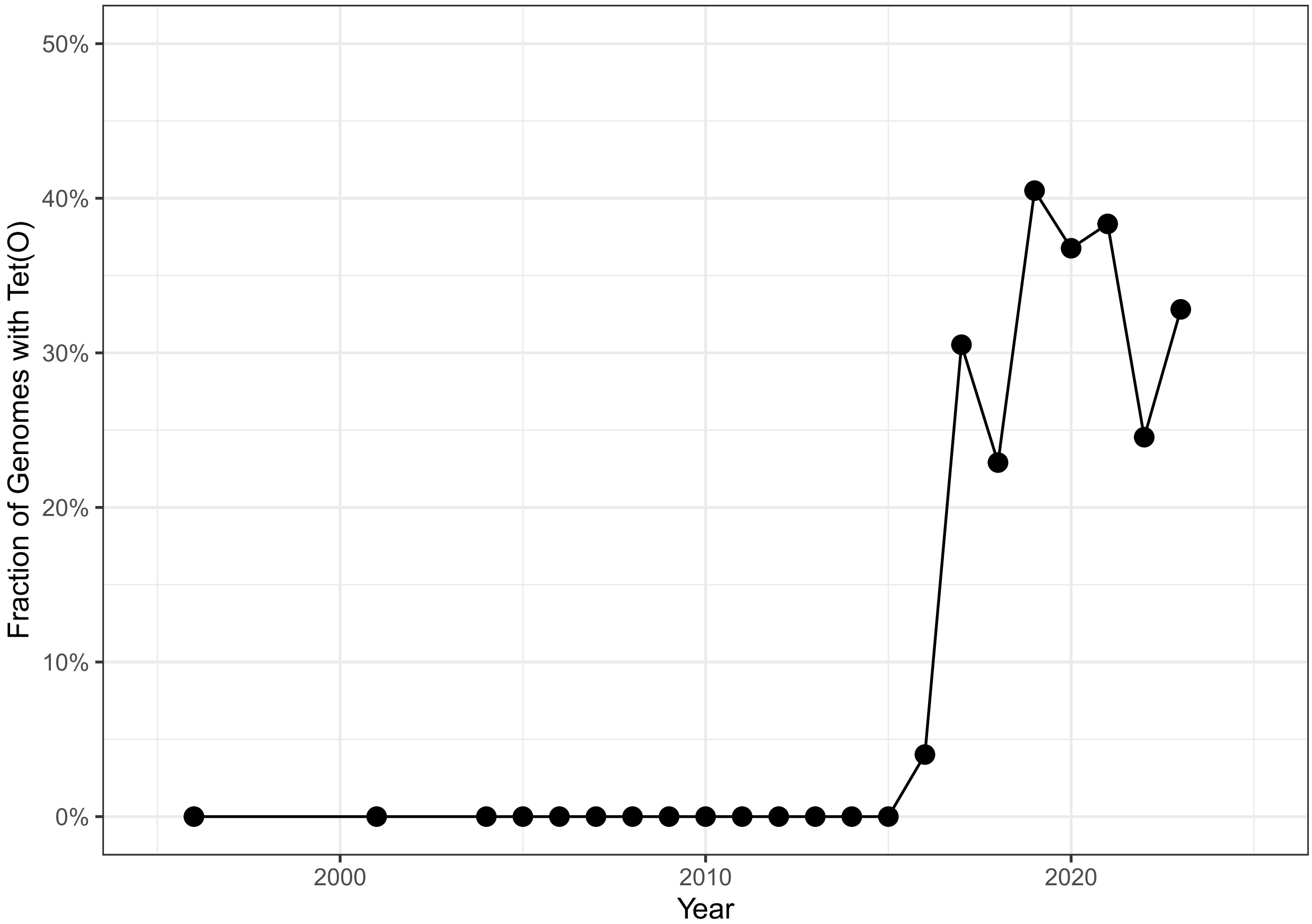

Supplementary Figure 2

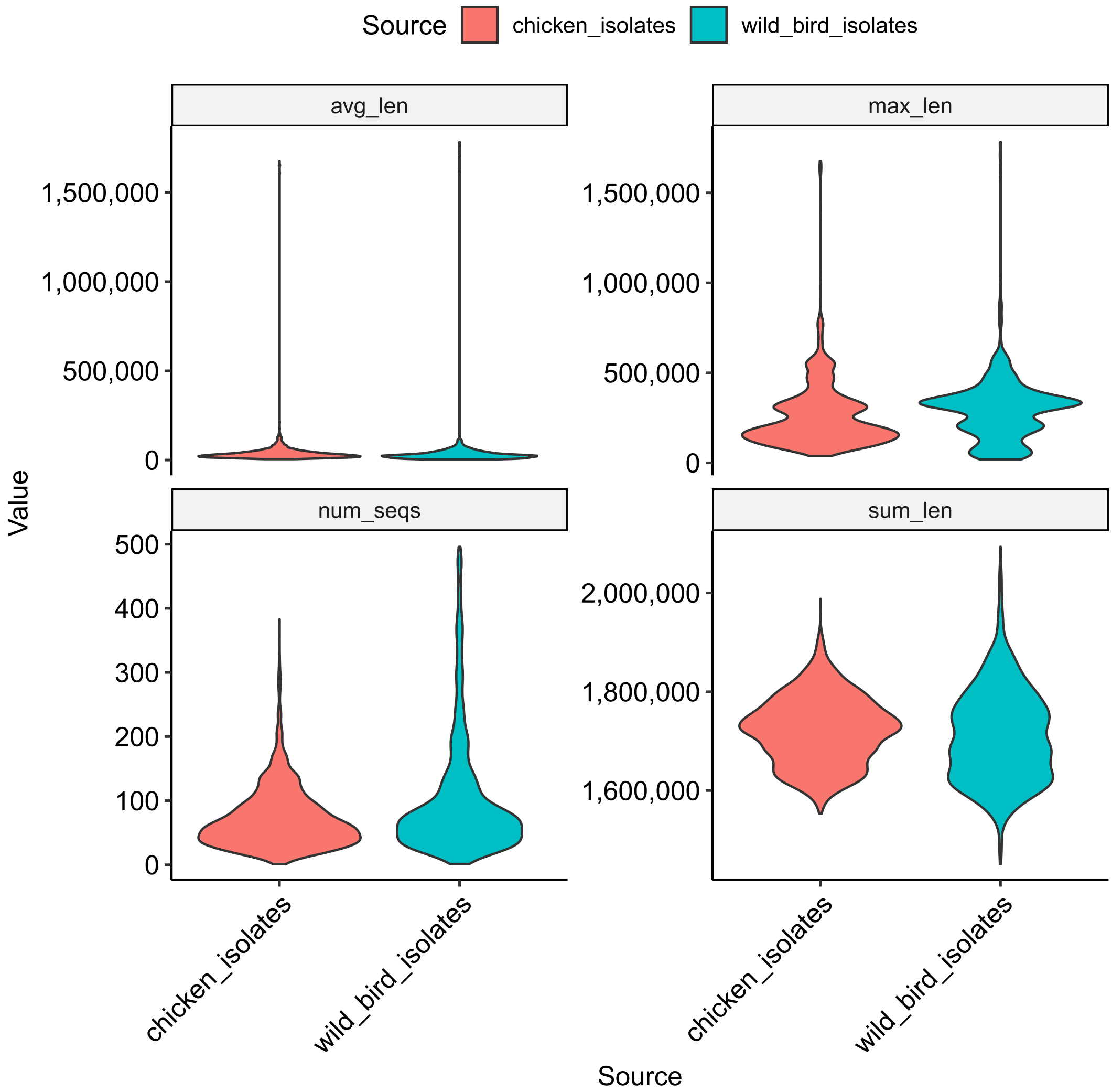

Supplementary Figure 3

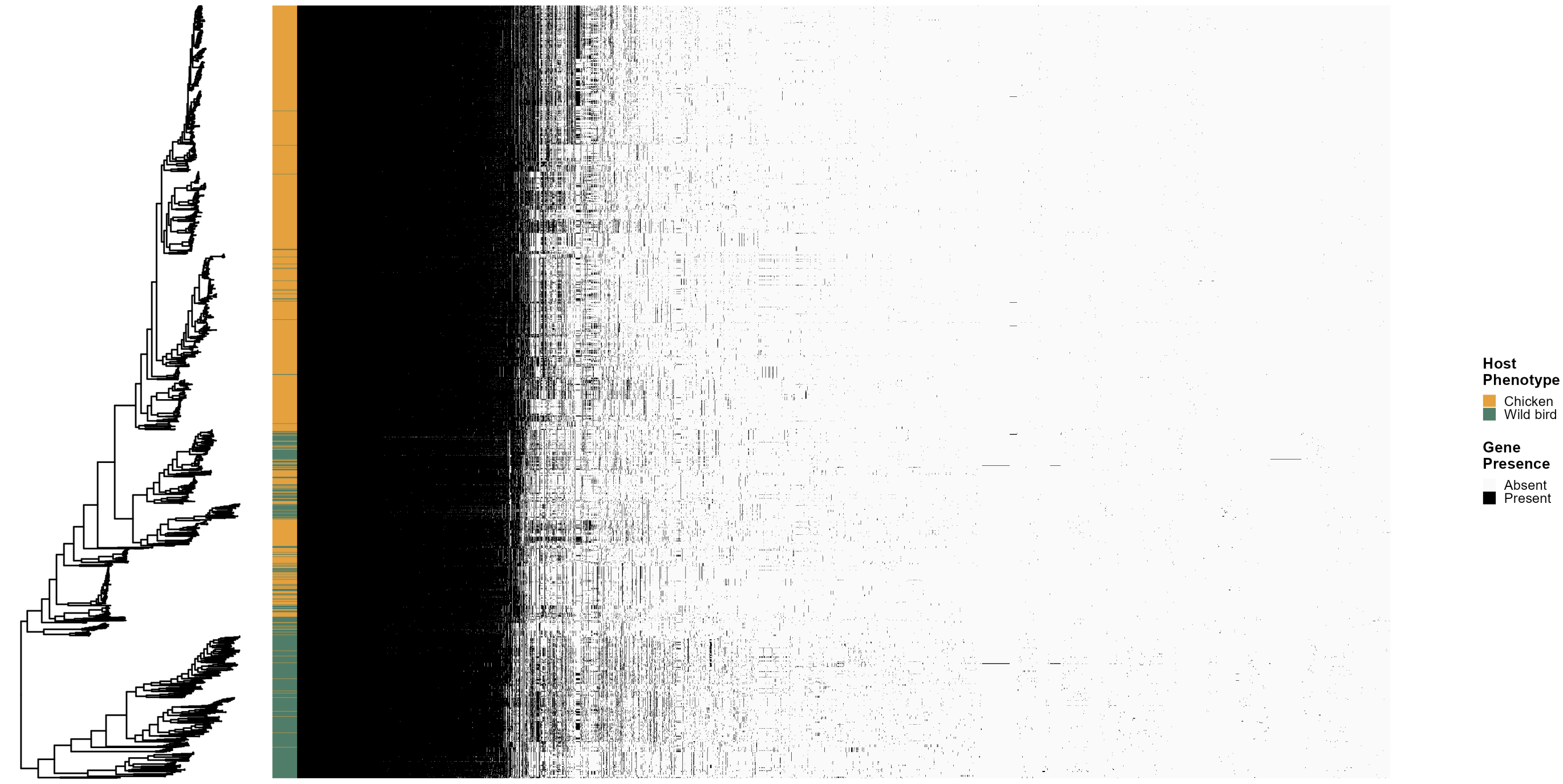

Supplementary Figure 4

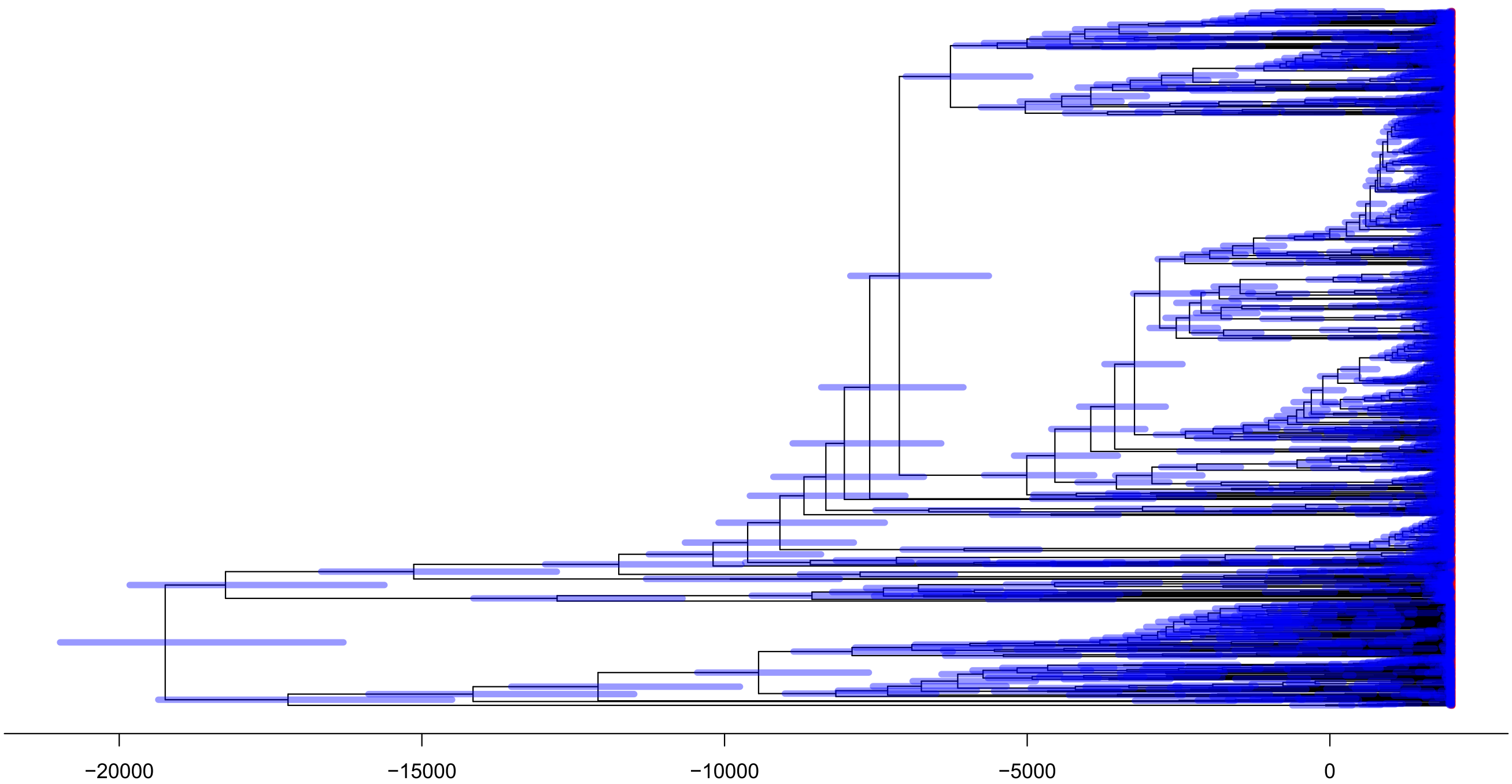

Supplementary Figure 5

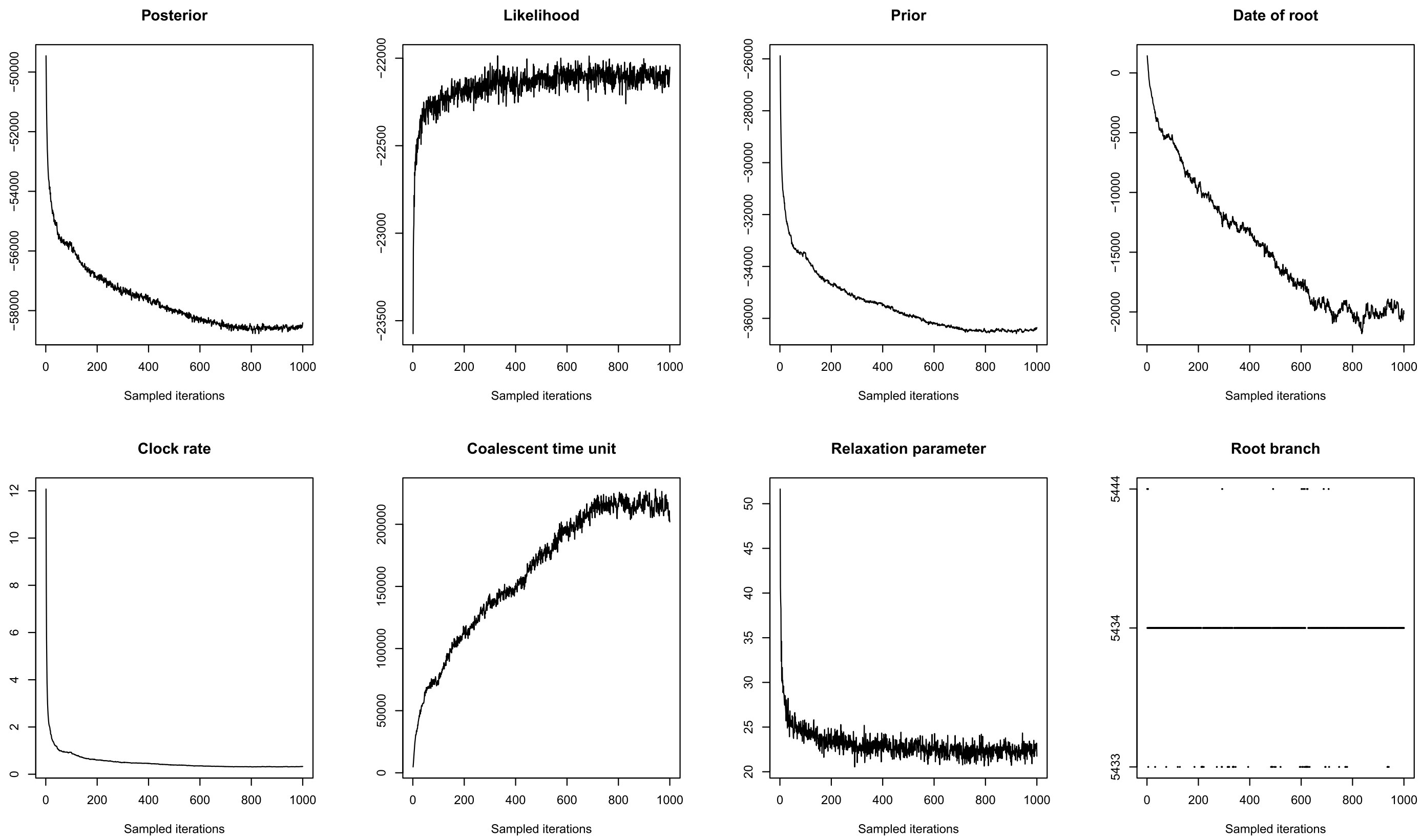

Supplementary Figure 6
